# Dysregulated insulin secretion is associated with pancreatic β‐cell hyperplasia and direct acinar‐β‐cell trans‐differentiation in partially eNOS‐deficient mice

**DOI:** 10.14814/phy2.15425

**Published:** 2022-08-19

**Authors:** Michela Novelli, Matilde Masini, Cecilia Vecoli, Stefania Moscato, Niccola Funel, Anna Pippa, Letizia Mattii, Chiara Ippolito, Daniela Campani, Danilo Neglia, Pellegrino Masiello

**Affiliations:** ^1^ Department of Translational Research and New Technologies in Medicine and Surgery University of Pisa Pisa Italy; ^2^ Institute of Clinical Physiology National Research Council (CNR) Pisa Italy; ^3^ Department of Clinical and Experimental Medicine University of Pisa Pisa Italy; ^4^ Interdepartmental Research Centre "Nutraceuticals and Food for Health" University of Pisa Pisa Italy; ^5^ Department of Surgical, Medical and Molecular Pathology, and Critical Care Medicine University of Pisa Pisa Italy; ^6^ Cardiovascular Department Fondazione Toscana Gabriele Monasterio per la Ricerca Medica e di Sanità Pubblica Pisa Italy

**Keywords:** eNOS+/− heterozygote mice, Notch‐1 pathway, pancreatic exocrine‐endocrine transdifferentiation, pancreatic β cells

## Abstract

eNOS‐deficient mice were previously shown to develop hypertension and metabolic alterations associated with insulin resistance either in standard dietary conditions (eNOS−/− homozygotes) or upon high‐fat diet (HFD) (eNOS+/− heterozygotes). In the latter heterozygote model, the present study investigated the pancreatic morphological changes underlying the abnormal glycometabolic phenotype. C57BL6 wild type (WT) and eNOS+/− mice were fed with either chow or HFD for 16 weeks. After being longitudinally monitored for their metabolic state after 8 and 16 weeks of diet, mice were euthanized and fragments of pancreas were processed for histological, immuno‐histochemical and ultrastructural analyses. HFD‐fed WT and eNOS+/− mice developed progressive glucose intolerance and insulin resistance. Differently from WT animals, eNOS+/− mice showed a blunted insulin response to a glucose load, regardless of the diet regimen. Such dysregulation of insulin secretion was associated with pancreatic β‐cell hyperplasia, as shown by larger islet fractional area and β‐cell mass, and higher number of extra‐islet β‐cell clusters than in chow‐fed WT animals. In addition, only in the pancreas of HFD‐fed eNOS+/− mice, there was ultrastructural evidence of a number of hybrid acinar‐β‐cells, simultaneously containing zymogen and insulin granules, suggesting the occurrence of a direct exocrine‐endocrine transdifferentiation process, plausibly triggered by metabolic stress associated to deficient endothelial NO production. As suggested by confocal immunofluorescence analysis of pancreatic histological sections, inhibition of Notch‐1 signaling, likely due to a reduced NO availability, is proposed as a novel mechanism that could favor both β‐cell hyperplasia and acinar‐β‐cell transdifferentiation in eNOS‐deficient mice with impaired insulin response to a glucose load.

## Graphical abstract

In heterozygous eNOS+/− mice, fed with either standard or high fat diet (HFD), blood insulin increase in response to a glucose load is impaired, combined with overt glucose intolerance and insulin resistance upon HFD. This pattern is associated with pancreatic β‐cell hyperplasia and, in HFD‐fed mice only, with ultrastructural evidence of hybrid acinar‐β cells, a likely result of a direct exocrine‐endocrine transdifferentiation process. Inhibition of Notch‐1 signalling documented in pancreatic cells is suggested as a putative molecular mechanism linking reduced NO availability with dysregulated pancreatic endocrine function and morphological changes.

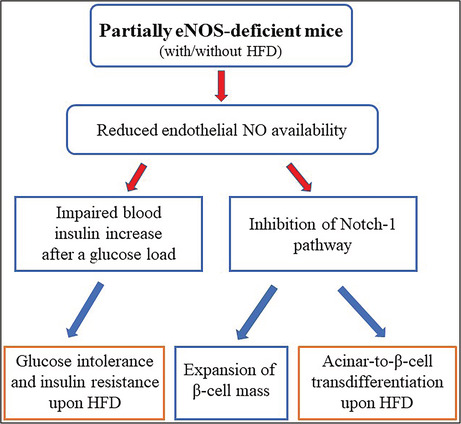

## 1 INTRODUCTION

Vascular endothelium has been increasingly recognized as a bidirectional pathogenetic actor linking vascular function and systemic metabolic regulation. Endothelial cells may be actively involved in the maintenance of metabolic homeostasis by different mechanisms including endothelial nitric oxide synthase (eNOS) function and NO availability (Pi et al., 2018). Clinical genetic studies have shown a strong association of eNOS polymorphisms with a number of metabolic and vascular abnormalities occurring in the metabolic syndrome and including insulin resistance, hypertriglyceridemia, low HDL cholesterol and hypertension (González‐Sánchez et al., 2007; Monti et al., 2003; Niu & Qi, 2011; Rittig et al., 2008; Vecoli et al., 2012), as well as with increased coronary artery disease risk (Hingorani et al., 1999). Indeed, endothelial NO production is anti‐atherogenic and modulates not only arterial pressure (Forte et al., 1997) but also insulin sensitivity by promoting delivery of substrate and insulin to skeletal muscle (Kapur et al., 1997; Roy et al., 1998). Consistently, eNOS null mice develop hypertension and insulin resistance (Duplain et al., 2001; Vecoli et al., 2014). Animals with partial eNOS gene deletion (+/− heterozygote mice), a model which more closely mimics the effects of clinical eNOS gene polymorphisms, have coronary vascular dysfunction and develop hypertension and overt insulin resistance mainly when fed with a high‐fat diet (HFD) (Cook et al., 2004; Vecoli et al., 2014).

Whether the primarily reduced NO availability occurring in eNOS +/− mice (Vecoli et al., 2014) as well as in some human polymorphisms (Rittig et al., 2008) may synergistically act with metabolic stress to modify endocrine pancreatic structure and function has not yet been fully elucidated (Broniowska et al., 2014; Sansbury & Hill, 2014). We hypothesized that reduced NO availability in eNOS+/− mice, alone and/or in combination with metabolic stress, could elicit abnormal morphofunctional changes in pancreatic cells, eventually affecting insulin secretion and glycometabolic homeostasis. Accordingly, in the present study we explored the histological, immunohistochemical, and ultrastructural features in the pancreas of partially knockout mice, fed with either a standard or a HFD, as compared with WT controls. The morphological patterns of pancreatic tissue were correlated with the longitudinal evaluation of glucose metabolic state and insulin secretory function in different experimental groups. In both chow‐ and HFD‐fed eNOS+/− mice, despite an impaired glucose‐stimulated insulin response, several features of β‐cell expansion were observed. Furthermore, and quite surprisingly, in the HFD‐fed eNOS+/− group only, ultrastructural evidence of hybrid exocrine/endocrine cells was found, suggesting the occurrence of a direct trans‐differentiation process of acinar to β cells in adult animals even in the absence of severe pancreatic damage. This finding deserves much attention and warrants further investigation to identify the underlying mechanisms, as there is always a great deal of interest in seeking new factors capable of influencing in vitro and in vivo reprogramming of pancreatic exocrine cells to β cells, as previously reported (Baeyens et al., 2005; Lemper et al., 2015; Miyazaki et al., 2016; Zhou et al., 2008). Based on these studies as reviewed by Aguayo‐Mazzucato & Bonner‐Weir (2018), we focused our attention on the possibility that modulation of Notch signaling could be involved in the pancreatic changes observed in our experimental model. The Notch signaling pathway is increasingly indicated among the crucial factors that not only influence developmental changes in multiple tissues and organs (Artavanis‐Tsakonas et al., 1999; Zhou et al., 2022), but also control homeostasis and differentiation in adult cells (Kopinke et al., 2012; Murtaugh et al., 2003; Zhou et al., 2022). It has indeed been shown that Notch signaling can regulate pancreatic islet function (Billiard et al., 2018) and act as gatekeeper of acinar‐to‐β‐cell conversion in vitro (Baeyens et al., 2009). In the present study we actually obtained preliminary evidence that a potential molecular mechanism for development of both β‐cell hyperplasia and acinar‐to‐β‐cell transdifferentiation might be linked to inhibition of Notch‐1 signaling, most likely determined by the reduced NO availability in partially eNOS‐deficient animals.

## 2 MATERIALS AND METHODS

### 2.1 Animals

Experiments were carried out in 4‐week‐old C57‐BL/6J wild type (WT) mice and eNOS+/− heterozygote mice from a previously established colony (Vecoli et al., 2014) in our facilities at the Institute of Clinical Physiology (IFC), National Research Council (CNR), Pisa. For each group, only male mice of 24–27 g body weight entered the protocol. Mice outside such weight range or showing signs of trauma or injury were excluded from the study. Starting at the age of 4 weeks, WT and eNOS+/− mice were fed for 16 weeks either a standard diet (chow food, CF) containing protein 23%, fat 5%, carbohydrate approximately 60%, fiber 5% and ash 5.5%, with 10% energy deriving from fat, or a high‐fat diet (HFD) containing protein 23%, fat 34%, carbohydrate approximately 30%, fiber 5% and ash 5.5%, with 60% energy deriving from fat. Both diets were purchased from Mucedola, Settimo Milanese, Italy. The animals were randomly assigned to each diet, thus being distributed into four groups: WT CF (*n* = 10); WT HFD (*n* = 8); eNOS+/− CF (*n* = 10) and eNOS+/− HFD (*n* = 10). Throughout the study period, the mice, housed in three or four per cage, remained under controlled temperature (21–23°C), 12/12 h light/dark cycle and were given food and water ad libitum. High‐fat food was renewed every day. Animal procedures were performed in strict compliance with protocols authorized by the Italian Ministry of Public Health and submitted to and approved by the local Ethical Committee of CNR.

### 2.2 Intraperitoneal glucose tolerance test (GTT)

After 8 and 16 weeks of CF or HFD, glucose (1.5 g/kg b.w. administered intraperitoneally [i.p.] as 16.5% solution) was given in the morning to 4‐h‐fasted conscious animals. Drops of blood were sequentially collected from the tail vein before and 15, 60, 120, and 180 min after the glucose injection and immediately used for glucose determination by glucometer. Some blood samples (at 0, 15 and 60 min) were also taken in EDTA‐treated tubes and centrifuged at 4°C to separate plasma that was stored at −20°C for subsequent insulin measurement. Insulin was determined by radioimmunoassay, as previously reported (Novelli et al., 2010).

### 2.3 Intraperitoneal insulin tolerance test (ITT)

After 8 and 16 weeks of diet, human insulin (0.75 U/kg b.w, Humulin R, Eli Lilly) was administered in the morning to 4‐h‐fasted conscious animals by intraperitoneal route. Blood glucose was measured by glucometer in drops of blood taken from the tail vein before and 15, 30, 60 and 120 min after the insulin injection.

### 2.4 Pancreas removal and extraction of total insulin content

After 16 weeks of HFD treatment, animals were anesthetized using pentobarbital (50 mg/kg b.w., i.p.) and killed by exsanguination before pancreas removal. Several pancreatic fragments were dissected and processed for histological and electron microscope analyses. The remaining pancreatic tissue (at least 80% of the total) was weighed and homogenized in 6 ml of cold acidified ethanol (0.7 M HCl/ethanol [1:3, vol/vol]) for extraction of insulin content. The homogenate was kept at 4°C for 24 h, and the supernatant obtained after centrifugation was stored at −20°C until assayed for determination of the extracted insulin.

### 2.5 Histological and immunohistochemical procedures

For morphological evaluation and measurement of β‐cell mass, suitable fragments of the pancreatic tail were fixed in 10% buffered formalin and processed using the standard procedure to obtain a paraffin‐embedded tissue block. Histological sections were prepared by routine methods and stained with hematoxylin–eosin (HE), as detailed elsewhere (Novelli et al., 2010). For immunohistochemical preparations, 2‐μm thick serial pancreatic sections were deparaffinized, dehydratated, and incubated with a guinea pig polyclonal anti‐insulin antibody (Zymed Laboratories). Immunoreactivity was visualized as described previously (Novelli et al., 2010). Laboratory personnel were blinded to treatment assignment.

Morphometric analysis was performed by a BX‐51 Olympus microscope connected to a computer by a color CCD camera. The analySIS^B^ software (Olympus) was used to acquire images at different magnifications. Islets were classified according to the size as large (L, >10,000 μm^2^), medium (M, 4000–10,000 μm^2^) or small (S, 500–4000 μm^2^). The relative fractional area of islets versus total pancreatic section area was determined on HE sections. The relative fractional area of β cells was established by dividing insulin‐positive stained area by total pancreatic section area. The β‐cell mass was estimated by multiplying insulin‐positive area fraction by pancreas weight (Bonner‐Weir, 2007). We considered groups of 2–5 cells immunostained with insulin and dispersed in the exocrine pancreas, as extra‐islet β‐cell clusters. Clusters were counted manually at 400× and their number per cm^2^ tissue was determined, as previously described (Wang et al., 2010). Immunofluorescence was performed on 5‐μm thick sections from paraffin‐embedded pancreas of control and eNOS+/− mice. Briefly, sections were dewaxed, rehydrated and antigen was retrieved by exposing sections to microwaves for 25 min at 600 W in 10 mM sodium citrate buffer at pH 6.0, containing 0.05% Tween 20. Then, aspecific binding sites were blocked by incubation of samples with a blocking solution (BS) (0.1% Tween, 0.25% BSA in PBS) for 1 h at room temperature (RT) and thereafter tissue sections were incubated overnight at 4°C with rabbit anti‐activated Notch‐1 primary antibody (Abcam, Cat# ab52627, RRID: AB_881725), diluted 1:50 in BS. After washings in BS, samples were incubated with Alexa Fluor® 488‐fluorescent goat anti‐rabbit secondary antibody (Abcam Cat# ab150077, RRID: AB_2630356), diluted 1:500 in BS and incubated for 90 min at RT in the dark. Finally, samples were mounted on slides with mounting medium (ProlongTM Diamond Antifade Mountant with DAPI, Thermo Fisher Scientific). Likewise, in order to detect hybrid cells in histological sections, a co‐immunofluorescence staining was performed using the rabbit polyclonal anti‐pancreatic α‐amylase antibody (Abcam Cat# ab21156, RRID: AB_446061) and the guinea pig polyclonal anti‐insulin antibody (BioRad Laboratories Inc, Italy, Cat# 5330‐0104G, RRID: AB_1605150,), diluted 1:200 and 1:250, respectively, in BS. Goat anti‐rabbit IgG (H + L) (Alexa Fluor® 488, code 111–545‐003, RRID: AB_2338046, Jackson ImmunoResearch) diluted 1:500 in BS, and donkey anti‐guinea pig IgG (H + L) (Alexa Fluor® 594, code 706–585‐148, RRID: AB_2340474, Jackson ImmunoResearch) diluted 1:200 in BS, were used as secondary antibodies. Negative controls were obtained by omitting the primary antibody and incubating the specimens with BS. Immunofluorescent images were captured under a confocal laser scanning microscopy (TC SSP8 Leica Microsystems) using 40× oil immersion lenses with 488 nm and 594 nm excitation wavelength lasers. Image analysis was performed in order to quantify immunofluorescence positivity for activated Notch‐1 in nuclei of pancreatic cells. In each microscopic field of the analyzed sections, reacting areas localized within the cell nuclei were quantified by CellSens Imaging Software (Olympus). Pixel color threshold (the minimum level at which nuclear areas were considered to be positive) was established on merged images, and the intensity of light blue pixels resulting from merging of bright blue (DAPI) and green (fluorescent‐positive) colors was evaluated. Data were expressed as the ratio between the immunofluorescent positive nuclear area and the total nuclear area.

### 2.6 Electron microscopy

Pancreatic samples were fixed with 2.5% (vol/vol) glutaraldehyde in 0.1 mmoL/L phosphate buffer, pH 7.4, for 2 h at 4°C, and then post‐fixed in 1% (vol/vol) phosphate‐buffered osmium tetroxide for 30 min at 4°C. Samples were dehydrated in a graded series of ethanol, transferred to propylene oxide and embedded in PolyBed 812 (Polysciences Inc.). Semi‐thin sections (500 nm thick) were placed on glass slides and stained with a 1:1 mixture of toluidine blue and methylene blue (both at 1% in bidistilled water). Ultrathin sections (60–80 nm thick) were cut with a diamond knife, placed on nickel grids (200 mesh), stained with uranyl acetate and lead citrate and observed with a Zeiss 902 electron microscope, as previously detailed (Novelli et al., 2010). Single or double immunogold labeling for α‐amylase and/or insulin was performed using the same antibodies employed for the co‐immunofluorescence analysis described above, together with protein A‐conjugated gold particles of different sizes (Agar Scientific). For the double immunogold technique, the labeling with anti‐α‐amylase antibody was applied on the reverse side of an ultrathin pancreatic section previously labeled with anti‐insulin antibody, according to Bendayan (Bendayan, 1982).

### 2.7 Statistical analysis

In the present study, data were processed utilizing GraphPad InStat 3.0 software (GraphPad Prism, RRID:SCR_002798) and are expressed as means ± standard error of the mean (SEM). Analysis of variance (ANOVA) with post hoc Tukey test was applied for data comparison among the four experimental mice groups. A *p* < 0.05, at least, was considered as significant. In addition, to confirm statistical significance for GTT and ITT data, repeated measures ANOVA, with Tukey test for multiple comparisons (IBM Corp. Released 2015. IBM SPSS Statistics for Windows, Version 23.0. Armonk, NY: IBM Corp.) was used.

## 3 RESULTS

### 3.1 Glucose tolerance tests after 8 and 16 weeks of CF or HFD

**FIGURE 1:**
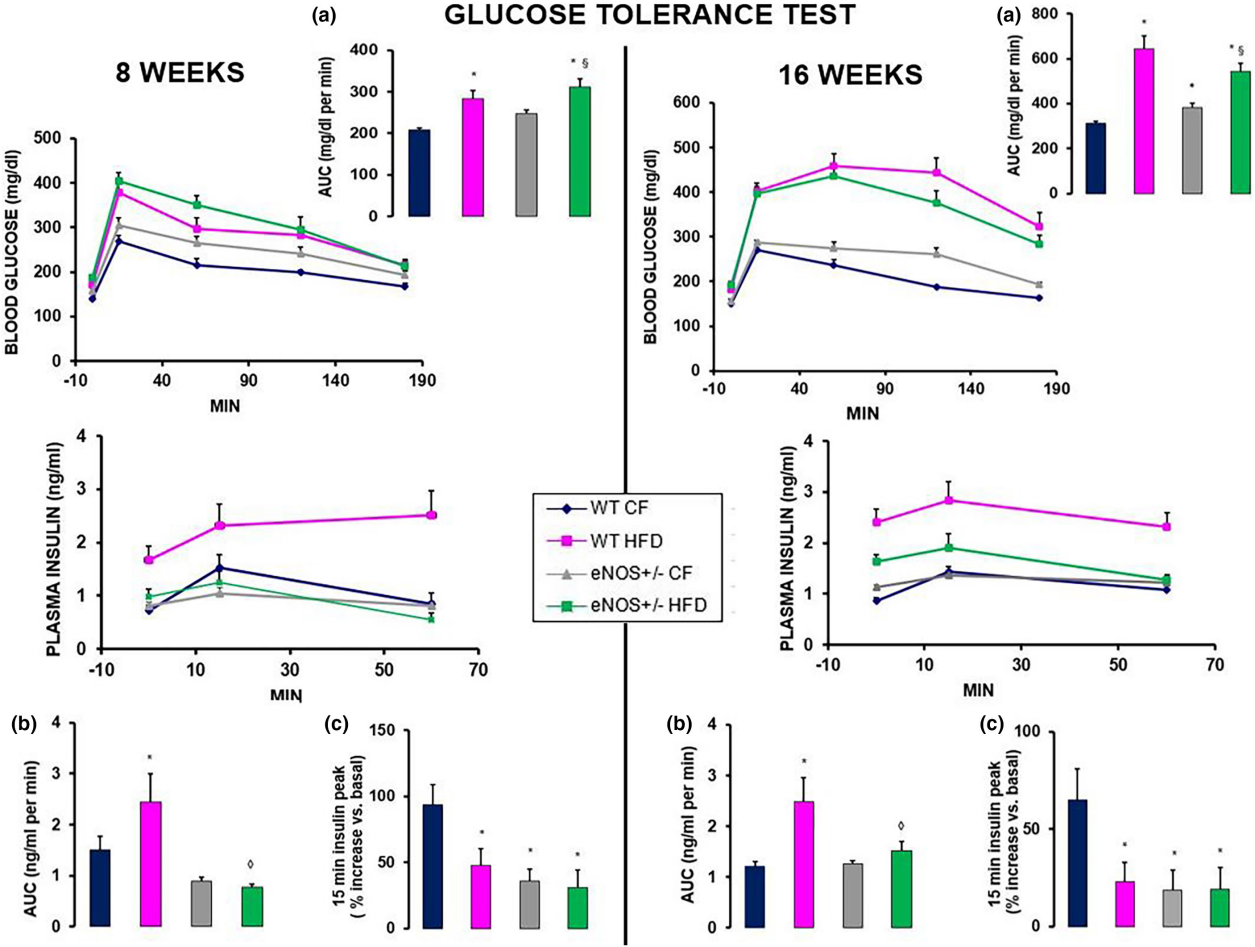
Intraperitoneal glucose tolerance tests in wild type and eNOS+/− mice, fed with either standard diet (chow‐fed, CF) or high‐fat diet (HFD). Glycemia and insulinemia were determined after glucose administration (1.5 g/kg i.p.), performed after 8 (left side) and 16 (right side) weeks of HFD diet. For glycemic values, the bar‐graph a in the insert shows the corresponding area under the curve (AUC). For insulinemic values, the bar‐graph b shows the corresponding AUC, while c shows the percent increase of 15‐min post‐loading plasma insulin level with respect to corresponding basal value. Mean ± SEM of 8–10 mice for each group. Repeated measures ANOVA assessed the significant effect of HFD on the post‐loading glycemic profiles in both genotypes (*p* < 0.01) and on the insulinemic profiles in WT animals only (*p* < 0.01). * *p* < 0.05, at least, versus WT CF; § *p* < 0.05, at least, versus eNOS+/− CF; ◊ *p* < 0.05, at least, versus WT HFD (ANOVA, post hoc Tukey test). WT CF, wild‐type chow‐fed mice; eNOS+/− CF, partial knockout eNOS chow‐fed mice; WT HFD, wild‐type HFD‐fed mice; eNOS+/− HFD, partial knockout eNOS HFD‐fed mice.

The intraperitoneal GTT, performed after 8 and 16 weeks of diet regimen, showed that HFD induced progressive glucose intolerance both in WT and eNOS+/− mice (Figure 1, upper left and right panels, inserts a). At 8 weeks, glycemic values and the corresponding areas under the curve (AUCs) were already significantly higher in HFD than in CF groups and further increased at 16 weeks. Similarly, basal glycemia was significantly higher at both 8 and 16 weeks in HFD vs CF groups (8 wks: WT CF 140 ± 3.7; WT HFD 171 ± 8.0*; eNOS CF 156 ± 6.5; eNOS HFD 187 ± 5.9*^§^ mg/dl; 16 wks: WT CF 151 ± 2.8; WT HFD 183 ± 5.8*; eNOS CF 156 ± 3.8; eNOS HFD 192 ± 8.4*^§^ mg/dl; *p < 0.01 vs WT CF, ^§^ *p* < 0.05, at least, vs eNOS CF [post‐hoc Tukey test]). Among CF groups, eNOS+/− mice had a slightly higher but not significantly different glycemic profile as compared to WT controls at both 8 and 16 weeks, yet with an AUC increase attaining statistical significance at 16 weeks. The plasma insulinemic profile during the glucose load (Figure 1, lower left and right panels, inserts b and c) showed further differences between WT and eNOS+/− animals (basal insulinemia was as follows: 8 wks, WT CF 0.72 ± 0.101; WT HFD 1.62 ± 0.286*; eNOS CF 0.70 ± 0.122; eNOS HFD 0.89 ± 0.161*^#^ ng/ml; 16 wks, WT CF 0.85 ± 0.073; WT HFD 2.41 ± 0.263*; eNOS CF 1.14 ± 0.040; eNOS HFD 1.63 ± 0.138*^#^ ng/ml; *p < 0.01 vs WT CF, ^#^ *p* < 0.05, at least, vs WT HFD [post‐hoc Tukey test]). In WT mice, HFD induced significantly higher basal and post‐loading plasma insulin levels than CF diet (with higher AUCs) at both 8 and 16 weeks, combined with a lower glucose‐stimulated insulin response, as shown by the smaller increase in insulin levels from 0 to 15 min (HFD vs CF WT: 49% vs 94% at 8 weeks, *p* < 0.05; 23% vs 65% at 16 weeks, *p* < 0.05). In eNOS+/− mice, HFD was associated with similar insulin levels at 8 and 16 weeks as compared to CF diet. Repeated measures ANOVA confirmed the significant effect of HFD on the post‐loading glycemic profiles in both genotypes (*p* < 0.01) and on the insulinemic profiles in WT animals only (*p* < 0.01). Notably, the insulin response to an acute glucose challenge was clearly depressed in eNOS+/− mice at both 8 and 16 weeks, regardless of the diet regimen, as indicated by the significantly lower (*p* < 0.05) post‐loading plasma insulin peak than in the WT CF group.

### 3.2 Insulin tolerance tests after 8 and 16 weeks of CF or HFD

**FIGURE 2:**
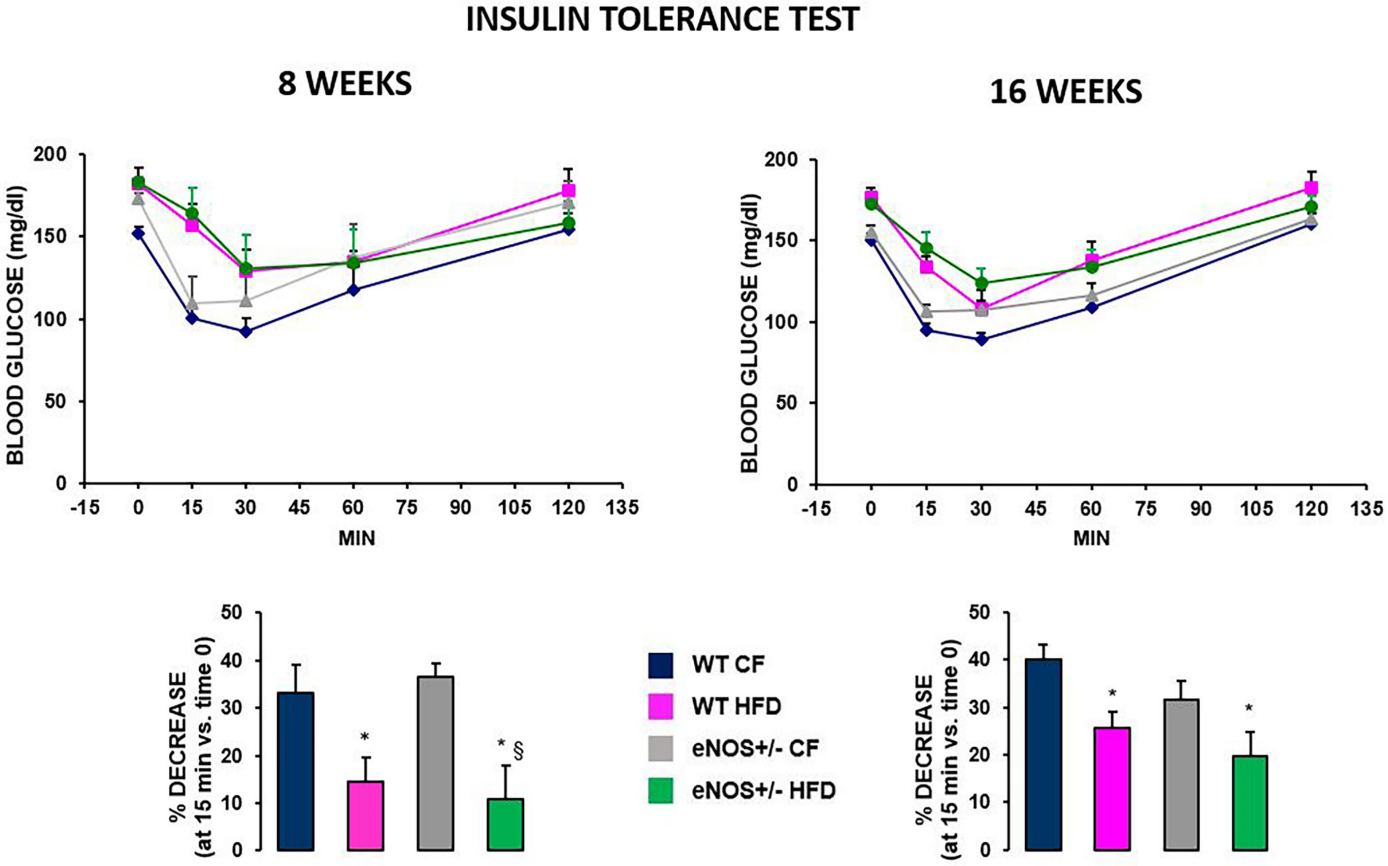
Insulin tolerance test in wild type and eNOS+/− mice, fed with either standard diet (CF) or high fat diet (HFD). Glycemia was determined before and after administration of human insulin (0.75 U/kg i.p.) after 8 (top left) and 16 weeks (top right) of HFD diet. Bar graphs at bottom show the percent decrease of 15‐min post‐loading blood glucose levels with respect to the corresponding basal values. Mean ± SEM of 8–10 mice for each group. Repeated measures ANOVA assessed the significant effect of HFD on the glycemic profiles in both genotypes at 16 weeks only (*p* < 0.05). * *p* < 0.05, at least, versus WT CF; § *p* < 0.01 versus eNOS+/− CF (ANOVA, post hoc Tukey test). Abbreviations are defined in Figure 1.

The intraperitoneal insulin tolerance tests performed after 8 and 16 weeks of CF or HFD in the four experimental animal groups showed no significant differences in insulin sensitivity between the two CF‐fed genotypes. (Figure 2). Upon HFD, basal glycaemic values in both WT and eNOS+/− groups were confirmed to be significantly higher than in the WT CF group (*p* < 0.05 at least) and dropped less after exogenous insulin administration, as shown by the significantly lower percent decrease of blood glucose levels at 15 min. Statistical evaluation by ANOVA for repeated measures of the overall glycemic profiles during ITT indicated that HFD regimen induced in both genotypes a progressive impairment in insulin sensitivity, as compared to CF groups, that attained statistical significance after 16 weeks.

### 3.3 Histology and immunohistochemistry of pancreatic tissue

**FIGURE 3:**
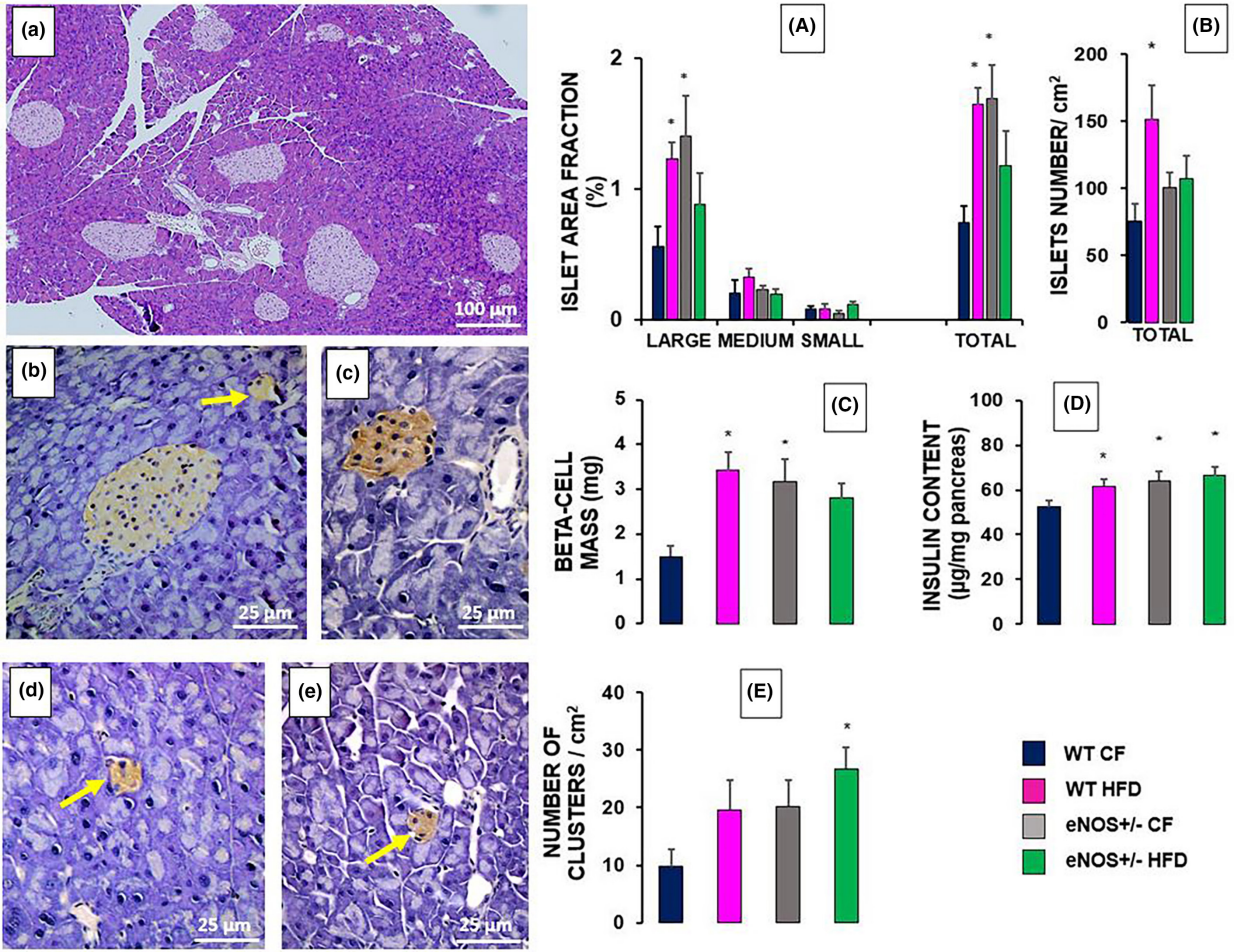
Fractional pancreatic islet area (A), islet number (B), β‐cell mass (C), insulin content (D) and number of extra‐islet β‐cell clusters (E) in WT and eNOS+/− mice. (a–e) Representative images of HE‐stained (a) or insulin immunostained (b–e) pancreatic sections. In a‐c, islets of various sizes can be observed in samples from WT HFD (a), eNOS+/− HFD (b) and eNOS+/− CF (c) groups. Fractional islet area, number of islets and of extra‐islet β‐cell clusters were calculated by a suitable morphometric software. The total islet area is expressed as a percent fraction of the whole pancreas section area and is also given according to the islet size (large, >10,000 μm^2^; medium, 4000–10,000 μm^2^; small, 500–4000 μm^2^). The β‐cell mass (mg) was obtained from the insulin area fraction multiplied by the pancreas weight. Pancreatic insulin content was measured after acid‐ethanol extraction of pancreatic fragments from 8–10 mice per group. We considered groups of 2–5 cells immunostained with insulin (b, d, e) as extra‐islet β‐cell clusters (yellow arrows). Most of these clusters can be also identified in HE stained section at high magnification (not shown). Mean ± SEM of 10–12 section measurements from at least four different mice per group. * *p* < 0.05, at least, versus WT CF (ANOVA, post hoc Tukey test).

The results of the morphometric analyses of HE‐stained and insulin immunostained sections of pancreatic tissue from the four experimental groups are reported in Figure 3 (panels A–C and E), together with a number of representative histological sections (a‐e). In the pancreas of HFD‐fed WT mice, islet fractional area (A), number of islets per area unit of the whole pancreas sections (B) and β‐cell mass (C) were significantly increased with respect to CF‐fed controls, consistent with adaptive β‐cell hyperplasia. Similar immunohistological patterns were also observed in eNOS‐deficient mice. In particular, in eNOS+/− CF animals, islet fractional area and β‐cell mass were significantly higher than in WT CF controls, with a minor gain in islet number. The same parameters were also increased, albeit not significantly (*p* = 0.056 for the β‐cell mass), in the eNOS+/− HFD mice as compared to WT CF group. When the fractional islet area was calculated according to the islet size, the differences among the experimental groups related mainly to the large islets rather than to the medium or small ones. The number of islets per area unit of the whole pancreas sections was found to be significantly increased only in the WT HFD mice, while there was just a trend to increase for both eNOS +/− groups. This means that the increase in islet fractional area and β‐cell mass in most cases is mainly due to increase in islet size, the contribution of increased islet number being weighty only in the WT HFD group. The total pancreatic insulin content, an additional reliable index of β‐cell mass in rodents (Novelli et al., 2007) was significantly increased in WT HFD mice and in both eNOS+/− groups when compared to the WT CF animals (Figure 3D). Interestingly, the number of extra‐islet β‐cell clusters (2–5 cells), considered an index of β‐cell neogenesis (Jetton et al., 2005), showed a trend to increase in WT HFD and eNOS+/− CF groups, and achieved a significant 2.5‐fold increase in eNOS+/− HFD mice (Figure 3E). The histological appearance of β‐cell clusters was similar in all the animal groups. Examples of islets of various sizes and clusters are given in representative HE‐stained and immunostained pancreatic sections of different mice groups (Figure 3a‐e). No preferential association of extra‐islet clusters with ductal structures was observed.

### 3.4 Electron microscopy of pancreatic tissue

**FIGURE 4:**
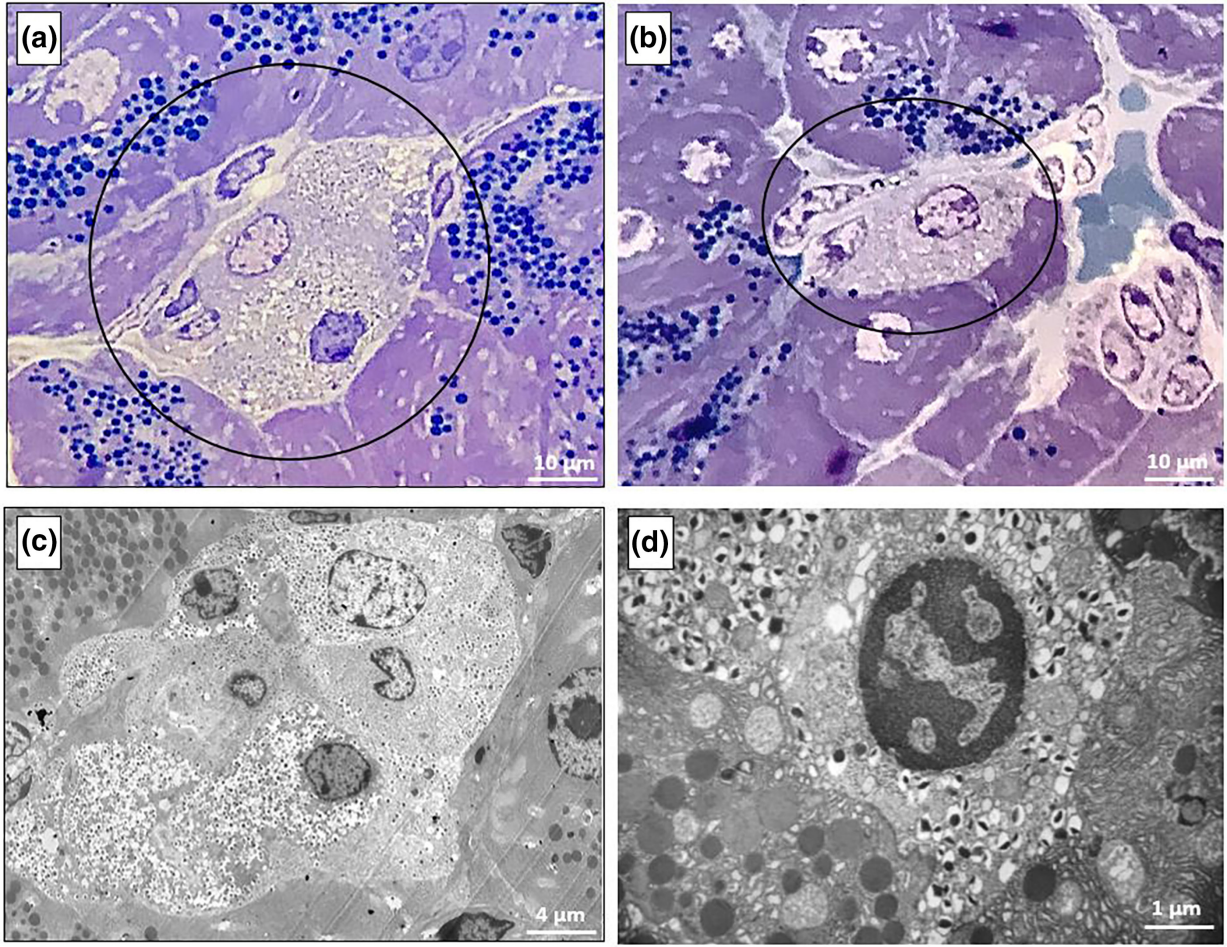
Representative toluidine blue/methylene blue‐stained semi‐thin pancreatic sections (a, b) and electron micrographs at low‐moderate magnification (c, d), showing extra‐islet β‐cell clusters surrounded by acinar cells. The β cells in the clusters (circled in a and b) contain mature insulin granules (dense core in a white halo) and are sandwiched between acinar cells, in which large zymogen granules are visible. Consecutive sections of the same pancreatic area (from a mouse of the eNOS+/− HFD group) are shown in a and c. Pancreatic sections in b and d are from mice of the WT CF and eNOS+/− CF group, respectively.

Semi‐thin pancreatic sections and subsequent electron microscopy (EM) analysis on consecutive ultrathin sections confirmed the presence of extra‐islet β‐cell clusters in all the four experimental groups. In Figure 4, representative images of these clusters are shown, consisting of few β cells filled with insulin granules and surrounded by exocrine acinar cells, characterized by the presence of large zymogen granules and a thick and voluminous rough endoplasmic reticulum (RER). Extra‐islet β cells are adjacent to acinar cells, but maintain clearly defined boundaries.

**FIGURE 5:**
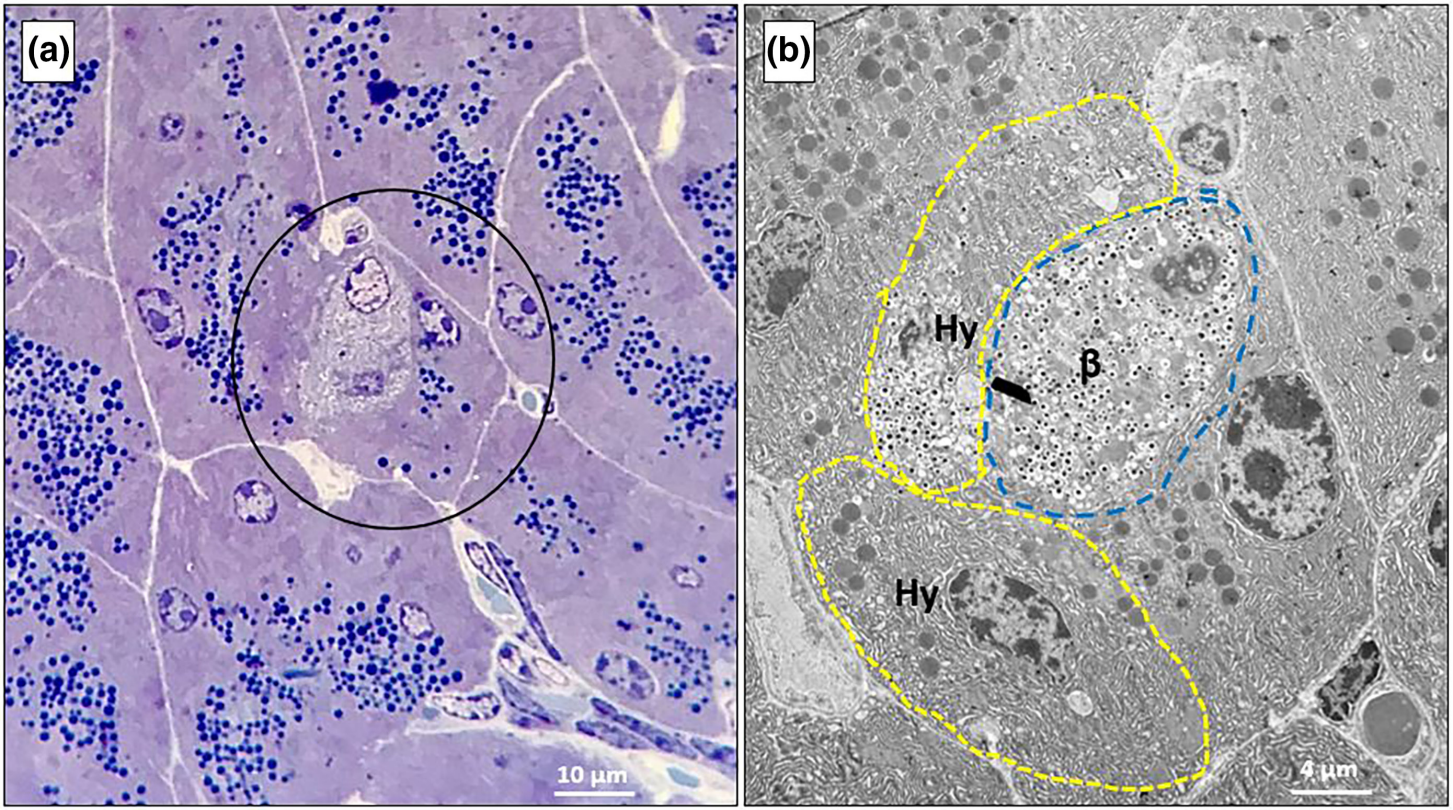
Representative toluidine blue/methylene blue‐stained semi‐thin pancreatic section (a**)** and low magnification electron micrograph (b) corresponding to the circled area in a, showing an extra‐islet β‐cell cluster with hybrid exocrine‐endocrine cells in a mouse of the eNOS+/− HFD group. In b, a β‐cell (β) and two hybrid cells (Hy) have been profiled with colored dashed lines to facilitate recognition.

**FIGURE 6:**
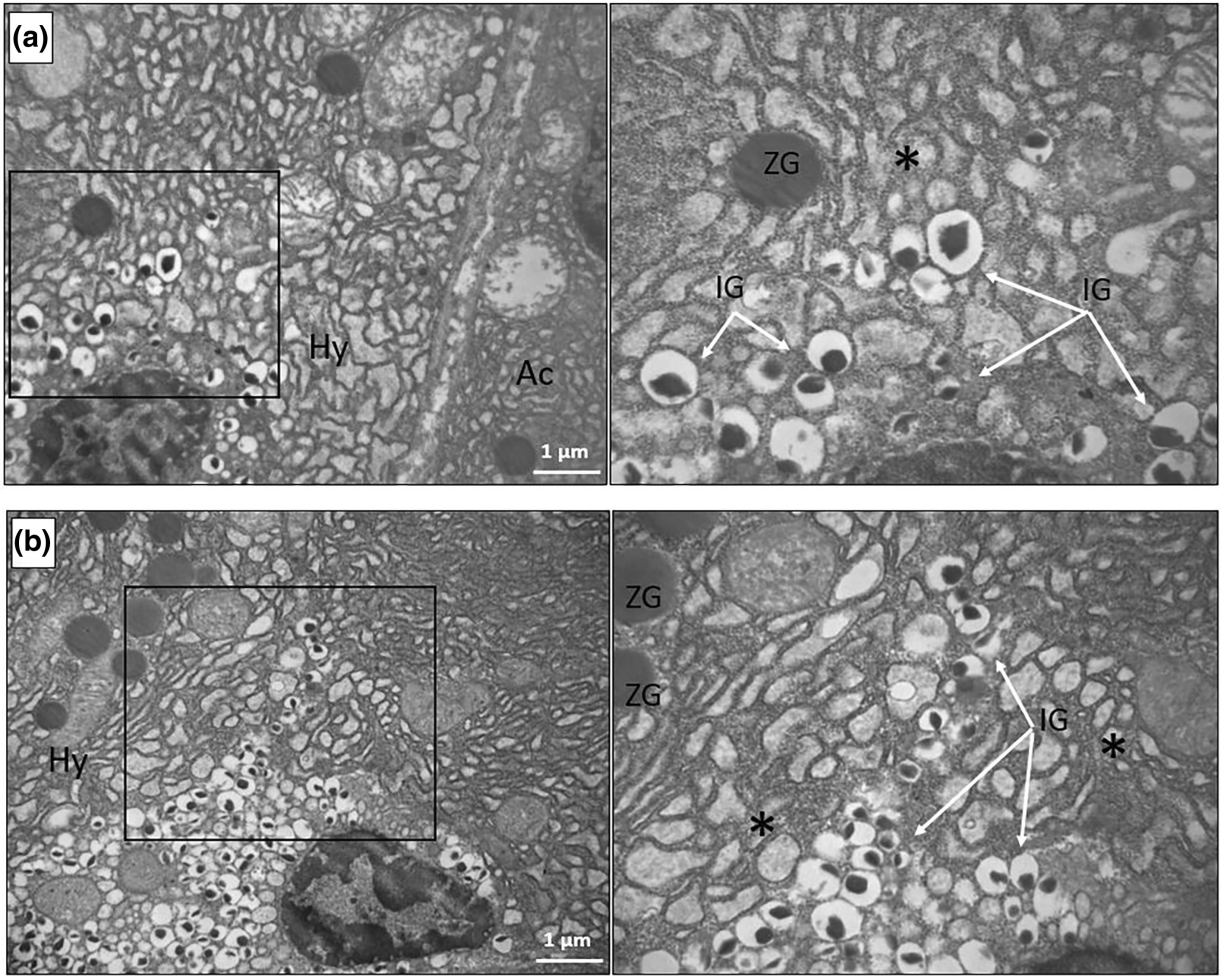
Electron micrographs of hybrid acinar‐β‐cells, showing the concomitant presence of insulin and zymogen granules in the pancreas of HFD‐fed eNOS+/− mice. (a) A hybrid cell (Hy) contains various insulin granules localized in proximity of the nucleus and is contiguous to a partially visible acinar cell (Ac), being the two cells clearly separated by their own plasma membranes. An enlargement (on the right) of the squared area shows several mature insulin granules (IG, white arrows) and a nearby zymogen granule (ZG) embedded in the thick RER (asterisk) typical of acinar cells. (b) Another hybrid cell from a different mouse of the same group, containing both zymogen and insulin granules, the latter again concentrated around the nucleus. In an enlargement (on the right) of the squared area, fully mature insulin granules (IG, white arrows) appear to sprout from the voluminous acinar‐type RER (asterisk) of the cell.

**FIGURE 7:**
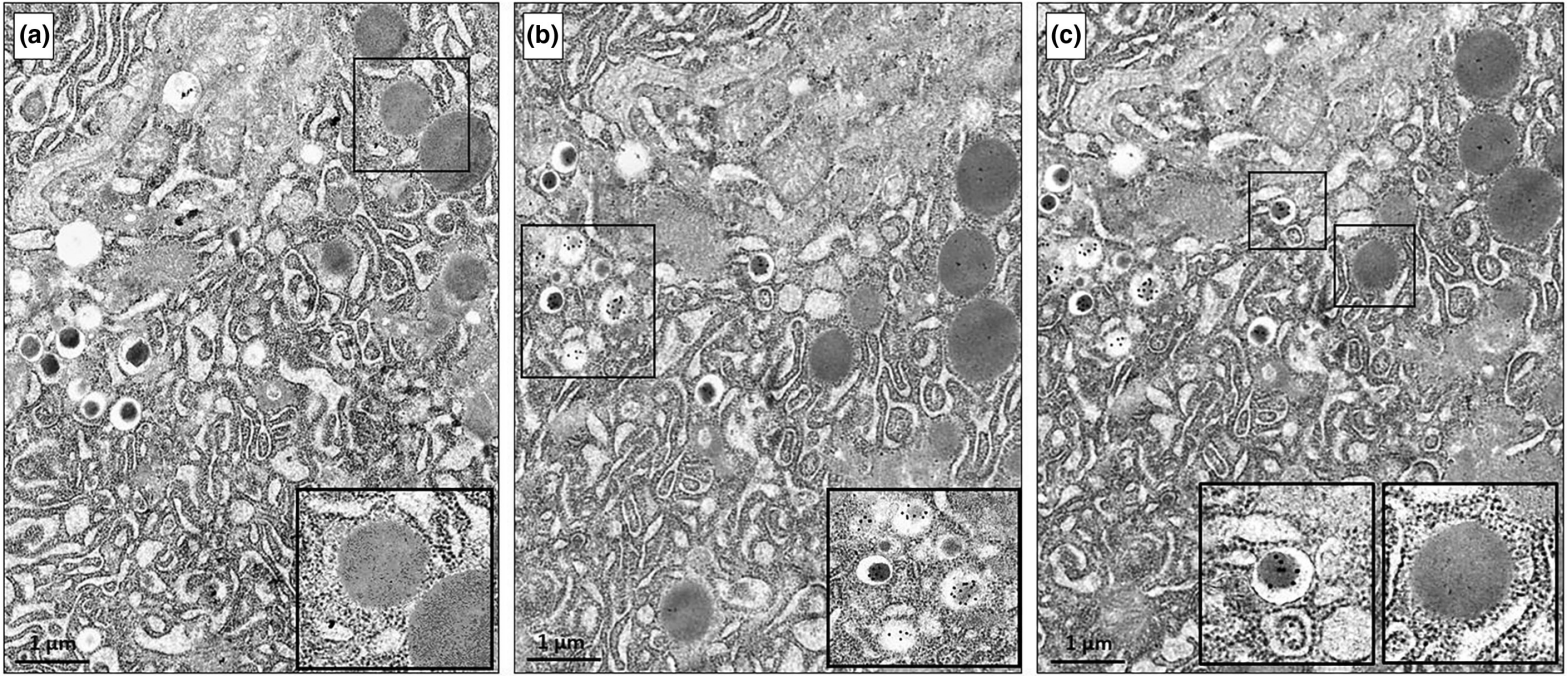
Electron micrographs of a hybrid acinar‐β‐cell from the pancreas of a HFD‐fed eNOS+/− mouse, labeled by single or double immuno‐gold technique (a–c). In each panel, the enlargement of a squared area highlights granule labeling. (a) Hybrid cell showing zymogen granules immunolabeled with 5 nm gold particles linked to anti‐α‐amylase antibodies, coexistent with unlabeled insulin granules. (b) Consecutive section of the same hybrid cell displaying insulin granules immunolabeled with 15 nm gold particles linked to anti‐insulin antibodies. (c) Double immunogold labeling using both anti‐insulin and anti‐α‐amylase antibodies shows the concomitant presence of specifically labeled insulin and zymogen granules. The additional anti‐α‐amylase immunolabeling was performed on the reverse side of the ultrathin section previously labeled with anti‐insulin antibody.

Unexpectedly, electron microscopy allowed to observe, in some of the clusters found in the pancreases of the HFD‐fed eNOS+/− mice, but not of the other groups, the presence of a number of hybrid cells containing both insulin and zymogen granules (Figure 5 at low and Figure 6 at high magnification). Hybrid cells were found in five of the six pancreases examined from the eNOS+/− HFD group. In Figure 5, a semi‐thin section (a) and a micrograph (b) corresponding to the same cluster area are shown. Thus, these clusters, although apparently similar to standard β‐cell clusters, contain one or more hybrid endocrine‐exocrine cells, as better evidenced by the high‐magnification images in Figures 6 and 7. Hybrid cells are usually separated from contiguous normal acinar cells by definite membrane boundaries (Figure 6a). They exhibit a variable number of mature insulin granules, often located in proximity of the nucleus (Figure 6a,b) and sometimes very close to zymogen granules (Figure 6a and Figure 7). At higher magnification, a number of insulin granules appear to sprout from the thick RER of the acinar cells (Figure 6a,b).

**FIGURE 8:**
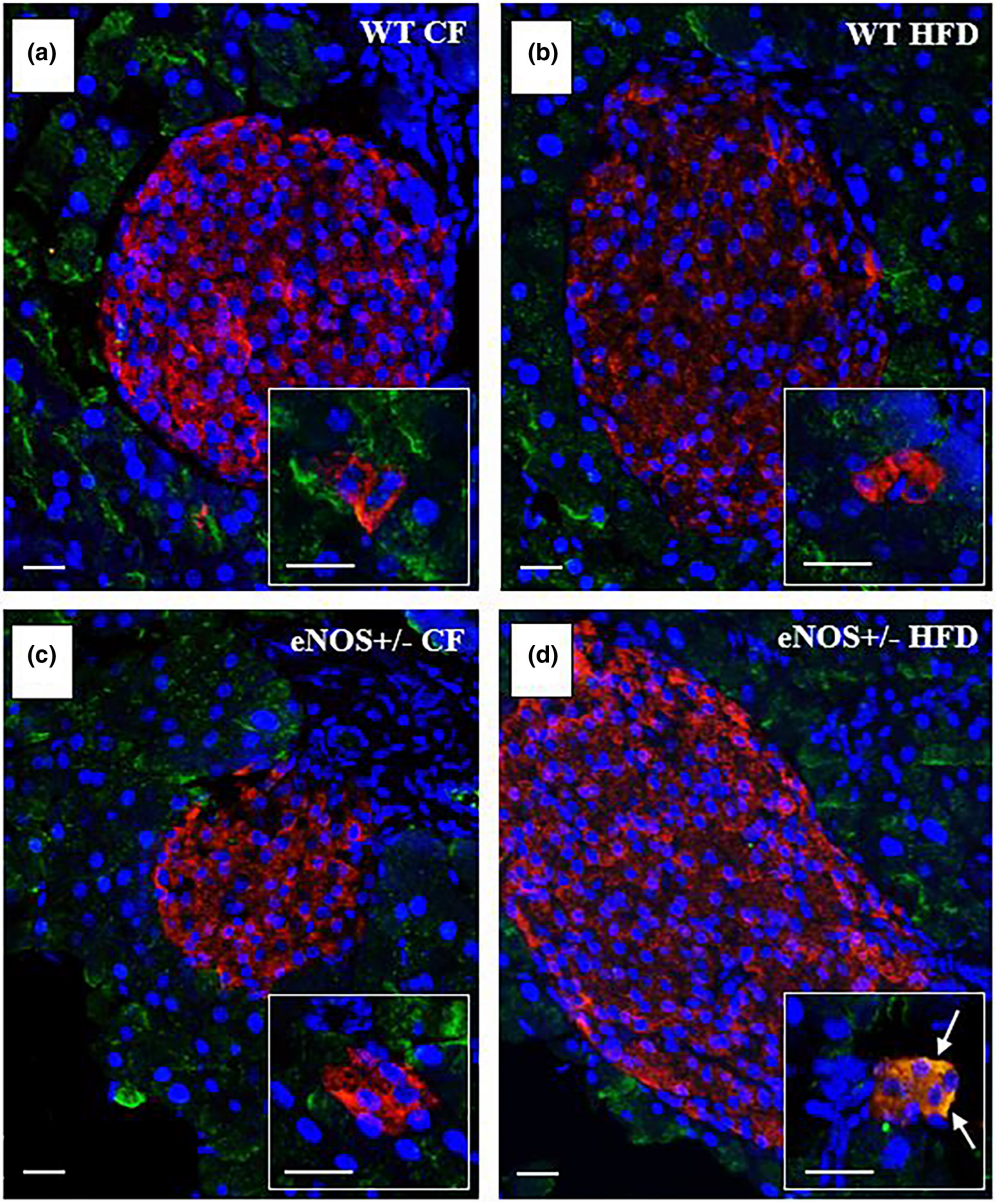
Confocal imaging of co‐immunofluorescence staining for α‐amylase and insulin to identify hybrid acinar‐β cells. (a–d) Representative images of pancreatic sections from the four different mice groups stained for α‐amylase (green), insulin (red), and DAPI (blue). Each square insert shows representative extra‐islet β‐cell clusters, identified in pancreatic sections from the same animal, with insulin and/or α‐amylase immunopositivity. In the insert of d, the yellow color (white arrows), resulting from the merge of green (α‐amylase) and red (insulin), attests the co‐localization of the two antigens in the cells of the cluster, thus revealing the presence of hybrid acinar‐β‐cells. Scale bars 20 μm.

Upon either single or double immunogold labeling of hybrid cells using appropriate anti‐α‐amylase and anti‐insulin antibodies, gold particles of different sizes identified either α‐amylase‐rich zymogen or insulin granules, according to the antibody specificity (Figure 7). Furthermore, we found that the presence of hybrid endocrine‐exocrine cells in the extra‐islet clusters could also be detected by co‐immunofluorescent staining using anti‐insulin and anti‐α‐amylase antibodies (Figure 8). Interestingly, yet with the limitation of a missing quantitative analysis, confocal imaging showed that hybrid acinar‐β‐cells could be revealed by the co‐localization (yellow color) of insulin (red) and α‐amylase (green) in the eNOS+/− HFD group only (Figure 8d) and not in the other experimental groups (Figure 8a–c). Hybrid cells were absent within the islets from any group. Further research is warranted to validate such co‐immunofluorescent staining as a reliable technique for rapid identification and quantification of hybrid cells in pancreatic sections.

### 3.5 Immunofluorescence analysis for Notch‐1 signaling in pancreatic tissue

**FIGURE 9:**
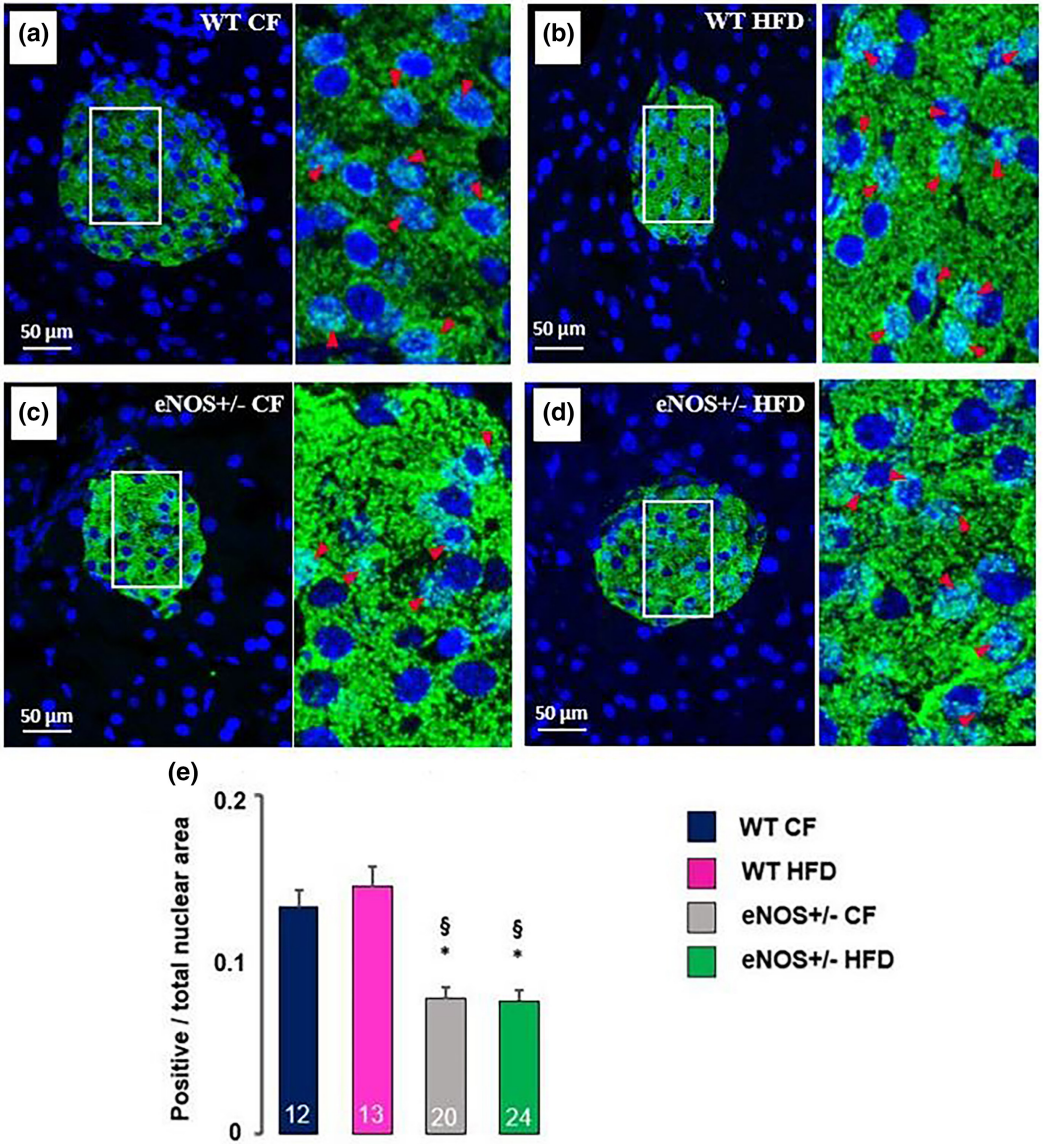
Immunofluorescence analysis of the nuclear localization of Notch‐1 intracellular domain (NICD) by confocal microscopy. In representative pancreatic sections from the four different mice groups, nuclear NICD green fluorescent staining appears more pronounced and frequent in WT (a, b) than in eNOS+/− (c, d) animals. Each image is flanked on the right side by a 3× magnification of the area bounded by the white lines, where distinctly fluorescent‐positive nuclei are indicated by magenta arrowheads. The ratio between fluorescent and total nuclear areas was quantitated by image analysis using the CellSens Imaging Software (Olympus) and shown in panel e. The total number of histological pancreatic sections, containing at least one pancreatic islet, examined from different individuals of the four animal groups (WT CF, *n* = 4; WT HFD, *n* = 4; eNOS+/− CF, *n* = 4; eNOS+/− HFD, *n* = 6), is indicated at the bottom of the bars in e (12 to 24 sections per group). * *p* < 0.01 versus WT CF; § *p* < 0.01 versus WT HFD (ANOVA, post hoc Tukey test). NICD, green; DAPI, blue.

In order to get some insights into the mechanisms involved in the observed changes in the pancreatic features of eNOS+/− mice (in particular β‐cell hyperplasia and acinar‐to‐β‐cell transdifferentiation), we used an immunofluorescent technique and confocal imaging to investigate the nuclear localization of Notch‐1 intracellular domain (NICD) as an index of Notch‐1 activation in the pancreatic tissue of the four animal groups. In Figure 9, the representative confocal images shown in panels a‐d and the bar graph in panel e summarizing the results of the quantitative analysis performed by a dedicated imaging software, reveal that the overall amount of nuclear NICD fluorescence (expressed as the ratio between positive over total nuclear area) is significantly reduced in the pancreatic islets of both CF‐ and HFD‐fed eNOS+/− mice, as compared to the WT groups. No significant fluorescence is observed in the exocrine pancreas, consistently with the reported weak expression of Notch‐1 in adult acinar cells in the mouse (Jensen et al., 2005).

## 4 DISCUSSION

In a previous work we showed that genetic eNOS deficiency in mice was associated with abnormal glucose metabolism and vascular coronary dysfunction, likely mediated by unbalance of cardiac insulin signaling pathways, with a shift toward a vasoconstrictive pattern (Vecoli et al., 2014). These findings occurred not only in homozygote eNOS−/−, but also in heterozygote eNOS+/− mice in either the absence or the presence of a high‐fat dietary regimen, which however additionally led to development of insulin resistance and overt diabetes. Taking into account that in human population polymorphic variants of the eNOS gene are common enough (González‐Sánchez et al., 2007; Vecoli et al., 2012), in the present study we aimed to better characterize the relationship between partial eNOS deficiency and dysregulation of glucose metabolism, particularly focusing on the so far unexplored morphological changes of the endocrine pancreas in this animal model.

Metabolic data obtained longitudinally after 8 and 16 weeks of either standard or high‐fat diet indicate that eNOS+/− genotype is characterized by progressive glucose intolerance and marked impairment of insulin responsiveness to a glucose load, regardless of the diet regimen. Upon standard diet, the glucose intolerance is mild/moderate, becoming more evident over time. At 16 weeks, eNOS+/− CF mice, as compared to WT controls, display normal glycemia at baseline but a higher AUC during GTT, associated with a small increase in basal plasma insulin levels and a reduced insulin secretory response to a glucose load. Upon administration of exogenous insulin, they show similar insulin sensitivity as WT mice. In both WT and eNOS+/− groups, HFD induces a progressive and marked glucose intolerance, with increased basal and post‐loading glycemia, as well as a reduction in insulin sensitivity at 8 and 16 weeks. However, HFD‐fed eNOS+/− mice have a significantly smaller increase in basal insulinemia than the corresponding WT animals, and a persistently blunted insulin secretion in response to glucose.

A major finding of the present study is actually the impaired insulin responsiveness to an acute glucose challenge in eNOS+/− mice, despite the concomitant presence of pancreatic β‐cell hyperplasia, as evaluated by histological, immunohistological and ultrastructural methods. In the eNOS+/− genotype, β‐cell hyperplasia encompasses increased β‐cell mass due to expansion of β‐cell area (more dependent on increase in islet size than in islet number) and enhanced number of extra‐islets β‐cell clusters, resulting in increased total pancreatic insulin content. Notably, in chow‐fed eNOS+/− mice, the observed increase in β‐cell mass and insulin content is similar to that reached by WT controls after 16 weeks of HFD. On the other hand, in eNOS+/− mice, HFD did not induce a further enhancement of β‐cell mass (even reduced by 12% as compared to the CF group), but caused a significant increase in the number of extra‐islet β‐cell clusters, which are considered to be expression of β‐cell neogenesis and perhaps the first core of β‐cell aggregation to form new islets (Jetton et al., 2005). Moreover, in the HFD‐fed eNOS+/− group, but not in the other groups, hybrid exocrine/endocrine cells, simultaneously containing zymogen and insulin granules, were detected by electron microscopy in most of the pancreases examined. These hybrid cells were surrounded by acinar cells and might represent an intermediate step of a direct exocrine‐endocrine transdifferentiation process, possibly responsible for the generation of extra‐islet β‐cell clusters. The occurrence of hybrid cells in the pancreas of the eNOS+/− HFD group was unexpected and hard to be quantified in the present study. We cannot exclude that hybrid acinar‐β cells might exist also in other groups. Apparently, their presence is associated to the combined influence of eNOS‐deficient genotype and HFD, as also confirmed by the preliminary results of a confocal immunofluorescence analysis that detected co‐localization of insulin and α‐amylase in the β‐cell clusters of the HFD‐fed eNOS+/− group only (Figure 8). Future investigations are required to definitely assess this concomitance and the underlying mechanisms.

### 4.1 Defective eNOS genotype, intra‐islet endothelial cells function and insulin secretion

The intra‐islet endothelial cells play a relevant role for maintaining an adequate β‐cell secretory activity, as repeatedly reported (Carlsson & Jansson, 2015; Hashimoto et al., 2015; Hogan & Hull, 2017; Stehouwer, 2018). An abnormal endothelial function may cause a mismatch between insulin release and blood flow adjustment in islet microcirculation, resulting in dysregulated insulin secretion. Actually, a complex endothelial‐β‐cell functional axis has been postulated in pancreatic islets (Burganova et al., 2021; Hogan & Hull, 2017; Narayanan et al., 2017; Peiris et al., 2014). Islet endothelial cells produce factors (e.g., hepatocyte growth factor or connective tissue growth factor), that enhance β‐cell differentiation and proliferation (Oliveira et al., 2018; Riley et al., 2015) and others that influence insulin biosynthesis and/or release, such as NO, endothelin‐1 and extracellular matrix molecules (Gregersen et al., 1996; Hogan & Hull, 2017; Johansson et al., 2009; Nikolova et al., 2006). Conversely, β‐cell‐derived factors (VEGF, insulin) impact islet endothelial function (Brissova et al., 2006). In particular, insulin binding to its receptors on endothelial cells activates the IRS/PI3K/Akt signaling pathway, promoting phosphorylation and activation of eNOS with consequent production of vasodilatory NO, which is crucial for the increment and maintenance of islet blood flow upon glucose stimulation (Carlsson & Jansson, 2015; Moldovan et al., 1996). The relevance of this insulin‐activated NO‐mediated vasodilatory pathway in the islet endothelial cells for ensuring the efficiency of β‐cell function is strengthened by the observation that disruption of endothelial cell‐specific insulin receptor substrate 2 (Irs2) in the ETIrs2KO mouse model causes a decrease in islet blood perfusion and defective glucose tolerance (Hashimoto et al., 2015). This impairment appears to be mainly related to inadequate blood flow‐dependent delivery of insulin from the islets into the systemic circulation, which is reversible if the islet blood perfusion is increased by an ACE inhibitor (Hashimoto et al., 2015).

Based on the described interaction between islet endothelial cells and β‐cell function, it is conceivable that the blunted insulin secretory responsiveness to a glucose load and the impaired glucose tolerance observed in the eNOS+/− mice in the present study could be at least in part due to a defective generation of NO by the islet endothelial cells and consequent reduction of islet microcirculatory vasodilation. In partial eNOS knockout mice we have previously observed a shift toward a vasoconstrictive pattern at the coronary vascular level, accounted for by an imbalance of the downstream insulin signaling (Vecoli et al., 2014). Such a mechanism could also occur in the islet vessels and be enhanced by the HFD‐induced dyslipidemia and obesity, which are able to further impair the Akt/eNOS signaling (De Boer et al., 2012; Vecoli et al., 2014; Wang et al., 2006). Thus, in chow‐fed and in particular in HFD‐fed eNOS+/− mice, the limited increase in post‐loading circulating insulin levels during GTT could be explained in part by an insufficient NO‐dependent vasodilation, capillary recruitment and insulin‐driven increase in blood flow in the islet vascular system, in analogy with the mechanism described in the muscle microcirculation (Clark et al., 2003; Kubota et al., 2011).

### 4.2 Defective eNOS genotype, β‐cell hyperplasia and Notch signaling

As expected, β‐cell hyperplasia developed in both HFD‐fed WT and, to a minor extent, eNOS+/− animals, most likely as a compensatory mechanism induced by the chronic metabolic surfeit and insulin resistance state (Alejandro et al., 2015; Jetton et al., 2005). Surprisingly, β‐cell hyperplasia was found even more evidently also in chow‐fed eNOS+/− mice, in the absence of HFD and insulin resistance. A possible explanation could be related to an enhanced stimulation of the endocrine pancreas by the post‐feeding hyperglycemia due to the impaired insulin responsiveness after glucose administration. However, it is unlikely that this temporary metabolic change would be sufficient to foster the observed adaptive β‐cell expansion without the contribution of other factors. It is also worth recalling that in the above‐mentioned model of endothelial cell‐specific Irs2 knockout mice, a defective insulin response to a glucose load is similarly associated with an increase in β‐cell mass or area (Hashimoto et al., 2015).

Further original and intriguing findings of the present study are the remarkable increase of extra‐islet β‐cell clusters in the pancreatic tissue of HFD‐fed eNOS+/− mice as well as the peculiar presence of hybrid exocrine‐endocrine cells, containing both zymogen and insulin granules in a cytoplasm quite typical of the acinar cell type. This morphological pattern is compatible with a direct acinar‐to‐β‐cell transdifferentiation process occurring upon metabolic stress, yet in the absence of overt damaging stimuli, at variance with similar observations of hybrid cells in a number of experimental and clinical conditions characterized by severe pancreatic damage (De Groef et al., 2015; Gu et al., 1997; Xu et al., 2008; Zhang et al., 2021). Interestingly, hybrid cells have also been found in the pancreas of a number of type 2 diabetic cadaveric donors, suggesting possible conversion from one cellular type to the other in association with the disease (Masini et al., 2017), that is, in a situation of metabolic derangement comparable in some respect with that occurring in the HFD‐fed animals of our study.

The occurrence of β‐cell hyperplasia and acinar‐to‐β‐cell conversion in association with the eNOS+/− genotype prompted us to presume that a common mechanism might be responsible for such concomitant change hypothetically concurring to increase the pancreatic potential for insulin production, even though the functional relevance of the transdifferentiation process is currently debated (Spears et al., 2021). On the basis of a number of studies aiming at identifying putative factors involved in β‐cell endogenous regeneration (Aguayo‐Mazzucato & Bonner‐Weir, 2018), we envisaged the possible involvement of Notch pathway in the eNOS+/− mouse model. Notch signaling, whose regulation is complex and not yet well understood (Zhou et al., 2022), is considered critical for cell‐fate determination during development (Artavanis‐Tsakonas et al., 1999; Zhou et al., 2022), including pancreatic endocrine and exocrine differentiation (Murtaugh et al., 2003), and is also required for survival, proliferation, and differentiation of adult cells (Bi & Kuang, 2015; Guruharsha et al., 2012; Kopinke et al., 2012). It is constitutively active in endothelial cells, including those of the pancreatic microvascular network, and in islet cells, with Delta‐like ligand‐1(Dll1) and ‐4 (Dll4) on neighboring cells serving as activating ligands (Billiard et al., 2018; Zhou et al., 2022), and is considered to play an important role in adult pancreatic islets homeostasis (Bartolome et al., 2019; Mirtschink & Chavakis, 2018; Rubey et al., 2020). Notably, the inhibition of Dll4‐Notch signaling was recently shown to drive differentiation of insulin‐producing cell progenitors, induce β‐cell proliferation and confer protection against streptozotocin‐induced diabetes (Billiard et al., 2018). Moreover, Notch inhibition has been reported to efficiently promote the conversion of rat acinar cell cultures to insulin‐producing β cells (Baeyens et al., 2009). In keeping with these indications, we have actually explored whether in partially deficient eNOS animals, Notch signaling pathway could be impaired in pancreatic tissue, contributing to the β‐cell mass expansion, the increase in extra‐islet β‐cell clusters and the acinar‐to‐β‐cell conversion observed in these animals. To preliminarily test this hypothesis, histological immunofluorescent methods using confocal microscopy were used to comparatively evaluate the presence of the active fragment of Notch‐1 protein, namely the Notch‐1 intracellular domain (NICD), in the nuclei of the pancreatic cells of the four experimental groups. The results indicate that in the eNOS+/− heterozygous mice, nuclear NICD is indeed decreased in islet cells as compared to the WT groups, regardless of the dietary regimen of the animals.

There are actually several lines of evidence linking Notch‐1 signaling with the eNOS‐NO pathway. Firstly, it has been shown that in aortic valve interstitial cells (AVICs), endothelial cell‐derived NO enhances the nuclear localization of NICD and regulates the expression of Notch‐ 1 target genes, thereby preventing aortic valve calcification (Bosse et al., 2013; Majumdar et al., 2021; Wang et al., 2021). Conversely, the reduced NO production, as occurs in endothelial cell dysfunction or in eNOS knockout mice, can in turn inhibit Notch‐1 signaling in AVICs and favor aortic valve calcification (Bosse et al., 2013; Garg, 2016). Actually, also in the eNOS+/− mouse a reduced availability of NO occurs, as assessed by the impairment in acetylcholine‐induced endothelium‐dependent vasodilation that we observed in this model (Vecoli et al., 2014). Notably, it appears that NO can regulate Notch‐1 signaling in AVICs by inducing S‐nitrosylation of USP9X, which results in stabilization of the E3 ubiquitin ligase MIB1 and potentiation of the ligand‐mediated Notch‐1 activation (Majumdar et al., 2021). At low NO concentrations, USP9X is not activated by S‐nitrosylation, leading to impairment of Notch‐1 ligand endocytosis and Notch‐1 signaling (Majumdar et al., 2021). Another mechanism of eNOS‐NO‐mediated Notch‐1 inhibition involves autophagy‐induced degradation of NICD‐1 (Ahn et al., 2016; Zhang et al., 2018). As elevated NO levels exert inhibitory effects on autophagy (Sarkar et al., 2011), it is plausible that the reduced availability of NO in eNOS+/− mice may favor induction of autophagic processes and hence Notch‐1 inhibition. Interestingly, in intermittent feeding, autophagy‐induced Notch‐1 degradation was found associated with enhanced pancreatic Ngn3 expression and β‐cell neogenesis (DiNicolantonio & McCarty, 2019).

Altogether, from these results and considerations it can be argued that partial eNOS deficiency is associated with and might be responsible for Notch‐1 inhibition in the micro‐environment of the pancreatic capillary network, thereby contributing to elicit β‐cell hyperplasia and acinar‐to‐β‐cell trans‐differentiation. However, further studies are certainly needed to confirm and extend our findings and assuredly establish by a molecular approach the involvement of Notch signaling as well as the role of metabolic stress in the observed pancreatic morphofunctional changes in the model of eNOS partially knockout mice.

## 5 CONCLUSIONS

The present study expands previous evidence of the existence of a causal link between endothelial dysfunction and abnormal glucose homeostasis involving endocrine pancreatic function (Vecoli et al., 2014). In an experimental genetic model of partial eNOS deficiency, insulin response to a glucose load was impaired, possibly due to an abnormal vasodilation capability of the pancreatic microcirculation. In addition, genetically modified mice developed β‐cell hyperplasia with preliminary evidence of an intriguing process of exocrine‐to‐β‐cell transdifferentiation as a putative consequence of the inhibition of Notch‐1 signaling in the pancreatic tissue, possibly linked to deficient NO production from the endothelial cells of the pancreatic microvascular network. There are obvious limitations in extending results obtained in a genetically modified mouse model to the clinical context. Nevertheless, the contributing role of a primary endothelial dysfunction in the development of glucose intolerance and eventually type 2 diabetes has been suggested by a number of human genetic investigations (Hingorani et al., 1999; Niu & Qi, 2011; Vecoli et al., 2012). The mechanisms suggested in the present experimental study need further validation and extension but might contribute to identify new targets of treatment to counteract the increasing prevalence of diabetes in the general population and its contribution as a residual risk of progressive atherosclerotic and myocardial diseases.

## AUTHOR CONTRIBUTIONS

D.N. and P.M. conceived and designed research; M.N., M.M., C.V., S.M., N.F., A.P., L.M., C.I., D.C., D.N., and P.M. performed experiments and analyzed data; M.N., M.M., C.V., S.M., N.F., D.C., D.N., and P.M. interpreted results of experiments; M.N., M.M., and S.M. prepared figures; P.M. and D.N. drafted manuscript; M.N., C.V., P.M., and D.N. edited and revised manuscript; All authors read and approved final version of the manuscript.

## FUNDING INFORMATION

The work was supported in part by Fondazione Toscana Gabriele Monasterio and IFC‐CNR internal funding and in part by University of Pisa internal funding.

## CONFLICT OF INTEREST

The authors have no conflict of interest to declare.

## References

[phy215425-bib-0001] Aguayo‐Mazzucato, C. , & Bonner‐Weir, S. (2018). Pancreatic β cell regeneration as a possible therapy for diabetes. Cell Metabolism, 27, 57–67. 10.1016/j.cmet.2017.08.007 28889951 PMC5762410

[phy215425-bib-0002] Ahn, J. S. , Ann, E. J. , Kim, M. Y. , Yoon, J. H. , Lee, H. J. , Jo, E. H. , Lee, K. , Lee, J. S. , & Park, H. S. (2016). Autophagy negatively regulates tumor cell proliferation through phosphorylation dependent degradation of the Notch1 intracellular domain. Oncotarget, 7, 79047–79063. 10.18632/oncotarget.12986 27806347 PMC5346697

[phy215425-bib-0003] Alejandro, E. U. , Gregg, B. , Blandino‐Rosano, M. , Cras‐Méneur, C. , & Bernal‐Mizrachi, E. (2015). Natural history of β‐cell adaptation and failure in type 2 diabetes. Molecular Aspects of Medicine, 42, 19–41. 10.1016/j.mam.2014.12.002 25542976 PMC4404183

[phy215425-bib-0004] Artavanis‐Tsakonas, S. , Rand, M. D. , & Lake, R. J. (1999). Notch signaling: cell fate control and signal integration in development. Science, 284, 770–776. 10.1126/science.284.5415.770 10221902

[phy215425-bib-0005] Baeyens, L. , Bonné, S. , Bos, T. , Rooman, I. , Peleman, C. , Lahoutte, T. , German, M. , Heimberg, H. , & Bouwens, L. (2009). Notch signaling as gatekeeper of rat acinar‐to‐β‐cell conversion in vitro. Gastroenterology, 136, 1750–1760. 10.1053/j.gastro.2009.01.047 19208356

[phy215425-bib-0006] Baeyens, L. , De Breuck, S. , Lardon, J. , Mfopou, J. K. , Rooman, I. , & Bouwens, L. (2005). In vitro generation of insulin‐producing beta cells from adult exocrine pancreatic cells. Diabetologia, 48, 49–57. 10.1007/s00125-004-1606-1 15616797

[phy215425-bib-0007] Bartolome, A. , Zhu, C. , Sussel, L. , & Pajvani, U. B. (2019). Notch signaling dynamically regulates adult β cell proliferation and maturity. The Journal of Clinical Investigation, 129, 268–280. 10.1172/JCI98098 30375986 PMC6307965

[phy215425-bib-0008] Bendayan, M. (1982). Double immunocytochemical labeling applying the protein A‐gold technique. The Journal of Histochemistry and Cytochemistry, 30, 81–85. 10.1177/30.1.6172469 6172469

[phy215425-bib-0009] Bi, P. , & Kuang, S. (2015). Notch signaling as a novel regulator of metabolism. Trends in Endocrinology and Metabolism, 26, 248–255. 10.1016/j.tem.2015.02.006 25805408 PMC4435535

[phy215425-bib-0010] Billiard, F. , Karaliota, S. , Wang, B. , Stellas, D. , Serafimidis, I. , Manousopoulou, A. , Koutmani, Y. , Ninou, E. , Golubov, J. , DaNave, A. , Tsakanikas, P. , Xin, Y. , Zhang, W. , Sleeman, M. , Yancopoulos, G. D. , Murphy, A. J. , Garbis, S. D. , Karalis, K. , & Skokos, D. (2018). Delta‐like ligand‐4‐Notch signaling inhibition regulates pancreatic islet function and insulin secretion. Cell Reports, 22, 895–904. 10.1016/j.celrep.2017.12.076 29386132

[phy215425-bib-0011] Bonner‐Weir, S. (2007). Beta‐cell turnover: Its assessment and implications. Diabetes, 50(Suppl 1), S20–S24. 10.2337/diabetes.50.2007.s20 11272192

[phy215425-bib-0012] Bosse, K. , Hans, C. P. , Zhao, N. , Koening, S. N. , Huang, N. , Guggilam, A. , LaHaye, S. , Tao, G. , Lucchesi, P. A. , Lincoln, J. , Lilly, B. , & Garg, V. (2013). Endothelial nitric oxide signaling regulates Notch1 in aortic valve disease. Journal of Molecular and Cellular Cardiology, 60, 27–35. 10.1016/j.yjmcc.2013.04.001 23583836 PMC4058883

[phy215425-bib-0013] Brissova, M. , Shostak, A. , Shiota, M. , Wiebe, P. O. , Poffenberger, G. , Kantz, J. , Chen, Z. , Carr, C. , Jerome, W. G. , Chen, J. , Baldwin, F. S. , Nicholson, W. , Bader, D. M. , Jetton, T. , Gannon, M. , & Powers, A. C. (2006). Pancreatic islet production of vascular endothelial growth factor‐A is essential for islet vascularization, revascularization, and function. Diabetes, 55, 2974–2985. 10.2337/db06-0690 17065333

[phy215425-bib-0014] Broniowska, K. A. , Oleson, B. J. , & Corbett, J. A. (2014). β‐Cell responses to nitric oxide. Vitamins and Hormones, 95, 299–322. 10.1016/B978-0-12-800174-5.00012-0 24559923

[phy215425-bib-0015] Burganova, G. , Bridges, C. , Thorn, P. , & Landsman, L. (2021). The role of vascular cells in pancreatic beta‐cell function. Frontiers in Endocrinology, 12, 667170. 10.3389/fendo.2021.667170 33981287 PMC8109179

[phy215425-bib-0016] Carlsson, P.‐O. , & Jansson, L. (2015). Disruption of insulin receptor signaling in endothelial cells shows the central role of an intact islet blood flow for in vivo β‐cell function. Diabetes, 64, 700–702. 10.2337/db14-1523 25713194

[phy215425-bib-0017] Clark, M. G. , Wallis, M. G. , Barret, E. J. , Vincent, M. A. , Richards, S. M. , Clerk, L. H. , & Ratting, S. (2003). Blood flow and muscle metabolism: a focus on insulin action. American Journal of Physiology. Endocrinology and Metabolism, 284, E241–E258. 10.1152/ajpendo.00408.2002 12531739

[phy215425-bib-0018] Cook, S. , Hugli, O. , Egli, M. , Ménard, B. , Thalmann, S. , Sartori, C. , Perrin, C. , Nicod, P. , Thorens, B. , Vollenweider, P. , Scherrer, U. , & Burcelin, R. (2004). Partial gene deletion of endothelial nitric oxide synthase predisposes to exaggerated high‐fat diet‐induced insulin resistance and arterial hypertension. Diabetes, 53, 2067–2072. 10.2337/diabetes.53.8.2067 15277387

[phy215425-bib-0019] De Boer, M. P. , Meijer, R. I. , Wijnstok, N. J. , Jonk, A. M. , Houben, A. J. , Stehouwer, C. D. , Smulders, Y. M. , Eringa, E. C. , & Serné, E. H. (2012). Microvascular dysfunction: a potential mechanism in the pathogenesis of obesity‐associated insulin resistance and hypertension. Microcirculation, 19, 5–18. 10.1111/j.1549-8719.2011.00130.x 21883642

[phy215425-bib-0020] De Groef, S. , Leuckx, G. , Van Gassen, N. , Staels, W. , Cai, Y. , Yuchi, Y. , Coppens, V. , De Leu, N. , Heremans, Y. , Baeyens, L. , Van de Casteele, M. , & Heimberg, H. (2015). Surgical injury to the mouse pancreas through ligation of the pancreatic duct as a model for endocrine and exocrine reprogramming and proliferation. Journal of Visualized Experiments, 102, e52765. 10.3791/52765 PMC4544398 26273954

[phy215425-bib-0021] DiNicolantonio, J. J. , & McCarty, M. (2019). Autophagy‐induced degradation of Notch1, achieved through intermittent fasting, may promote beta cell neogenesis: Implications for reversal of type 2 diabetes. Open Heart, 6, e001028. 10.1136/openhrt-2019-001028 31218007 PMC6546199

[phy215425-bib-0022] Duplain, H. , Burcelin, R. , Sartori, C. , Cook, S. , Egli, M. , Lepori, M. , Vollenweider, P. , Pedrazzini, T. , Nicod, P. , Thorens, B. , & Scherrer, U. (2001). Insulin resistance, hyperlipidemia, and hypertension in mice lacking endothelial nitric oxide synthase. Circulation, 104, 342–345. 10.1161/01.cir.104.3.342 11457755

[phy215425-bib-0023] Forte, P. , Copland, M. , Smith, L. M. , Milne, E. , Sutherland, J. , & Benjamin, N. (1997). Basal nitric oxide synthesis in essential hypertension. Lancet, 349, 837–842. 10.1016/S0140-6736(96)07631-3 9121259

[phy215425-bib-0024] Garg V. Notch signaling in aortic valve development and disease. In Nakanishi T , Markwald RR , Baldwin HS , Keller BB , Srivastava D , Yamagishi H (Eds.), Etiology and morphogenesis of congenital heart disease: From gene function and cellular interaction to morphology (pp. 371–376). Springer; 2016. 29787042

[phy215425-bib-0025] González‐Sánchez, J. L. , Martínez‐Larrad, M. T. , Sáez, M. E. , Zabena, C. , Martínez‐Calatrava, M. J. , & Serrano‐Ríos, M. (2007). Endothelial nitric oxide synthase haplotypes are associated with features of metabolic syndrome. Clinical Chemistry, 53, 91–97. 10.1373/clinchem.2006.075176 17110473

[phy215425-bib-0026] Gregersen, S. , Thomsen, J. L. , Brock, B. , & Hermansen, K. (1996). Endothelin‐1 stimulates insulin secretion by direct action on the islets of Langerhans in mice. Diabetologia, 39, 1030–1035. 10.1007/BF00400650 8877285

[phy215425-bib-0027] Gu, D. , Arnush, M. , & Sarvetnick, N. (1997). Endocrine/exocrine intermediate cells in streptozotocin‐treated Ins‐IFN‐gamma transgenic mice. Pancreas, 15, 246–250. 10.1097/00006676-199710000-00005 9336787

[phy215425-bib-0028] Guruharsha, K. G. , Kankel, M. W. , & Artavanis‐Tsakonas, S. (2012). The Notch signalling system: recent insights into the complexity of a conserved pathway. Nature Reviews. Genetics, 13, 654–666. 10.1038/nrg3272 PMC4369923 22868267

[phy215425-bib-0029] Hashimoto, S. , Kubota, N. , Sato, H. , Sasaki, M. , Takamoto, I. , Kubota, T. , Nakaya, K. , Noda, M. , Ueki, K. , & Kadowaki, T. (2015). Insulin receptor substrate‐2 (Irs2) in endothelial cells plays a crucial role in insulin secretion. Diabetes, 64, 876–886. 10.2337/db14-0432 25277391

[phy215425-bib-0030] Hingorani, A. D. , Liang, C. F. , Fatibene, J. , Lyon, A. , Monteith, S. , Parsons, A. , Haydock, S. , Hopper, R. V. , Stephens, N. G. , O'Shaughnessy, K. M. , & Brown, M. J. (1999). A common variant of the endothelial nitric oxide synthase (Glu298‐Asp) is a major risk factor for coronary artery disease in the UK. Circulation, 100, 1515–1520. 10.1161/01.cir.100.14.1515 10510054

[phy215425-bib-0031] Hogan, M. F. , & Hull, R. L. (2017). The islet endothelial cell: A novel contributor to beta cell dysfunction in diabetes. Diabetologia, 60, 952–959. 10.1007/s00125-017-4272-9 28396983 PMC5505567

[phy215425-bib-0032] Jensen, J. N. , Cameron, E. , Garay, M. V. , Starkey, T. W. , Gianani, R. , & Jensen, J. (2005). Recapitulation of elements of embryonic development in adult mouse pancreatic regeneration. Gastroenterology, 128, 728–741. 10.1053/j.gastro.2004.12.008 15765408

[phy215425-bib-0033] Jetton, T. L. , Lausier, J. , LaRock, K. , Trotman, W. E. , Larmie, B. , Habibovic, A. , Peshavaria, M. , & Leahy, J. L. (2005). Mechanisms of compensatory beta‐cell growth in insulin‐resistant rats: Roles of Akt kinase. Diabetes, 54, 2294–2304. 10.2337/diabetes.54.8.2294 16046294

[phy215425-bib-0034] Johansson, A. , Lau, J. , Sandberg, M. , Borg, L. A. , Magnusson, P. U. , & Carlsson, P. O. (2009). Endothelial cell signalling supports pancreatic beta cell function in the rat. Diabetologia, 52, 2385–2394. 10.1007/s00125-009-1485-6 19669728

[phy215425-bib-0035] Kapur, S. , Bedard, S. , Marcotte, B. , Cote, C. H. , & Marette, A. (1997). Expression of nitric oxide synthase in skeletal muscle: A novel role for nitric oxide as a modulator of insulin action. Diabetes, 46, 1691–1700. 10.2337/diab.46.11.1691 9356014

[phy215425-bib-0036] Kopinke, D. , Brailsford, M. , Pan, F. C. , Magnuson, M. A. , Wright, C. V. E. , & Murtaugh, L. C. (2012). Ongoing Notch signalling maintains phenotypic fidelity in the adult exocrine pancreas. Developmental Biology, 362, 57–64. 10.1016/j.ydbio.2011.11.010 22146645 PMC3254730

[phy215425-bib-0037] Kubota, T. , Kubota, N. , Kumagai, H. , Yamaguchi, S. , Kozono, H. , Takahashi, T. , Inoue, M. , Itoh, S. , Takamoto, I. , Sasako, T. , Kumagai, K. , Kawai, T. , Hashimoto, S. , Kobayashi, T. , Sato, M. , Tokuyama, K. , Nishimura, S. , Tsunoda, M. , Ide, T. , … Kadowaki, T. (2011). Impaired insulin signaling in endothelial cells reduces insulin‐induced glucose uptake by skeletal muscle. Cell Metabolism, 13, 294–307. 10.1016/j.cmet.2011.01.018 21356519

[phy215425-bib-0038] Lemper, M. , Leuckx, G. , Heremans, Y. , German, M. S. , Heimberg, H. , Bouwens, L. , & Baeyens, L. (2015). Reprogramming of human pancreatic exocrine cells to beta‐like cells. Cell Death and Differentiation, 22, 1117–1130. 10.1038/cdd.2014.193 25476775 PMC4572860

[phy215425-bib-0039] Majumdar, U. , Manivannan, S. , Basu, M. , Ueyama, Y. , Blaser, M. C. , Cameron, E. , McDermott, M. R. , Lincoln, J. , Cole, S. E. , Wood, S. , Aikawa, E. , Lilly, B. , & Garg, V. (2021). Nitric oxide prevents aortic valve calcification by S‐nitrosylation of USP9X to activate NOTCH signaling. Science Advances, 7, eabe3706. 10.1126/sciadv.abe3706 33547080 PMC7864581

[phy215425-bib-0040] Masini, M. , Marselli, L. , Himpe, E. , Martino, L. , Bugliani, M. , Suleiman, M. , Boggi, U. , Filipponi, F. , Occhipinti, M. , Bouwens, L. , De Tata, V. , & Marchetti, P. (2017). Co‐localization of acinar markers and insulin in pancreatic cells of subjects with type 2 diabetes. PLoS One, 12, e0179398. 10.1371/journal.pone.0179398 28617859 PMC5472296

[phy215425-bib-0041] Mirtschink, P. , & Chavakis, T. (2018). The missed Notch to bring down diabetes. Trends in Endocrinology and Metabolism, 29, 448–450. 10.1016/j.tem.2018.03.011 29606343

[phy215425-bib-0042] Miyazaki, S. , Tashiro, F. , & Miyazaki, J. (2016). Transgenic expression of a single transcription factor Pdx1 induces transdifferentiation of pancreatic acinar cells to endocrine cells in adult mice. PLoS One, 11, e0161190. 10.1371/journal.pone.0161190 27526291 PMC4985130

[phy215425-bib-0043] Moldovan, S. , Livingston, E. , Zhang, R. S. , Kleinman, R. , Guth, P. , & Brunicardi, F. C. (1996). Glucose‐induced islet hyperemia is mediated by nitric oxide. American Journal of Surgery, 171, 16–20. 10.1016/s0002-9610(99)80066-x 8554133

[phy215425-bib-0044] Monti, L. D. , Barlassina, C. , Citterio, L. , Galluccio, E. , Berzuini, C. , Setola, E. , Valsecchi, G. , Lucotti, P. , Pozza, G. , Bernardinelli, L. , Casari, G. , & Piatti, P. (2003). Endothelial nitric oxide synthase polymorphisms are associated with type 2 diabetes and the insulin resistance syndrome. Diabetes, 52, 1270–1275. 10.2337/diabetes.52.5.1270 12716763

[phy215425-bib-0045] Murtaugh, L. C. , Stanger, B. Z. , Kwan, K. M. , & Melton, D. A. (2003). Notch signaling controls multiple steps of pancreatic differentiation. Proceedings of the National Academy of Sciences of the United States of America, 100, 14920–14925. 10.1073/pnas.2436557100 14657333 PMC299853

[phy215425-bib-0046] Narayanan, S. , Loganathan, G. , Dhanasekaran, M. , Tucker, W. , Patel, A. , Subhashree, V. , Mokshagundam, S. , Hughes, M. G. , Williams, S. K. , & Balamurugan, A. N. (2017). Intra‐islet endothelial cell and beta‐cell crosstalk: Implication for islet cell transplantation. World Journal of Transplantation, 7, 117–128. 10.5500/wjt.v7.i2.117 28507914 PMC5409911

[phy215425-bib-0047] Nikolova, G. , Jabs, N. , Konstantinova, I. , Domogatskaya, A. , Tryggvason, K. , Sorokin, L. , Fässler, R. , Gu, G. , Gerber, H. P. , Ferrara, N. , Melton, D. A. , & Lammert, E. (2006). The vascular basement membrane: A niche for insulin gene expression and beta cell proliferation. Developmental Cell, 10, 397–405. 10.1016/j.devcel.2006.01.015 16516842

[phy215425-bib-0048] Niu, W. , & Qi, Y. (2011). An updated meta‐analysis of endothelial nitric oxide synthase gene: three well‐characterized polymorphisms with hypertension. PLoS One, 6, e24266. 10.1371/journal.pone.0024266 21912683 PMC3166328

[phy215425-bib-0049] Novelli, M. , Bonamassa, B. , Masini, M. , Funel, N. , Canistro, D. , De Tata, V. , Martano, M. , Soleti, A. , Campani, D. , Paolini, M. , & Masiello, P. (2010). Persistent correction of hyperglycemia in streptozotocin‐nicotinamide‐induced diabetic mice by a non‐conventional radical scavenger. Naunyn‐Schmiedeberg's Archives of Pharmacology, 382, 127–137. 10.1007/s00210-010-0524-7 PMC2904902 20512314

[phy215425-bib-0050] Novelli, M. , D'Aleo, V. , Lupi, R. , Paolini, M. , Soleti, A. , Marchetti, P. , & Masiello, P. (2007). Reduction of oxidative stress by a new low‐molecular‐weight antioxidant improves metabolic alterations in a nonobese mouse diabetes model. Pancreas, 35, e10–e17. 10.1097/mpa.0b013e318150e4f2 18090226

[phy215425-bib-0051] Oliveira, A. G. , Araújo, T. G. , Carvalho, B. , Rocha, G. Z. , Santos, A. , & Saad, M. J. A. (2018). The Role of hepatocyte growth factor (HGF) in insulin resistance and diabetes. Front Endocrinol (Lausanne), 9, 503. 10.3389/fendo.2018.00503 30214428 PMC6125308

[phy215425-bib-0052] Peiris, H. , Bonder, C. S. , Coates, P. T. H. , Keating, D. J. , & Jessup, C. F. (2014). The β‐cell/EC axis: How do islet cells talk to each other? Diabetes, 63, 3–11. 10.2337/db13-0617 24357688

[phy215425-bib-0053] Pi, X. , Xie, L. , & Patterson, C. (2018). Emerging roles of vascular endothelium in metabolic homeostasis. Circulation Research, 123, 477–494. 10.1161/CIRCRESAHA.118.313237 30355249 PMC6205216

[phy215425-bib-0054] Riley, K. G. , Pasek, R. C. , Maulis, M. F. , Peek, J. , Thorel, F. , Brigstock, D. R. , Herrera, P. L. , & Gannon, M. (2015). Connective tissue growth factor modulates adult b‐cell maturity and proliferation to promote b‐cell regeneration in mice. Diabetes, 64, 1284–1298. 10.2337/db14-1195 25392241 PMC4375083

[phy215425-bib-0055] Rittig, K. , Holder, K. , Stock, J. , Tschritter, O. , Peter, A. , Stefan, N. , Fritsche, A. , Machicao, F. , Häring, H. U. , & Balletshofer, B. (2008). Endothelial NO‐synthase intron 4 polymorphism is associated with disturbed in vivo nitric oxide production in individuals prone to type 2 diabetes. Hormone and Metabolic Research, 40, 13–17. 10.1055/s-2007-1004527 18095216

[phy215425-bib-0056] Roy, D. , Perreault, M. , & Marette, A. (1998). Insulin stimulation of glucose uptake in skeletal muscles and adipose tissues in vivo is NO dependent. The American Journal of Physiology, 274, E692–E699. 10.1152/ajpendo.1998.274.4.E692 9575831

[phy215425-bib-0057] Rubey, M. , Chhabra, N. F. , Gradinger, D. , Sanz‐Moreno, A. , Lickert, H. , Przemeck, G. K. H. , & Hrabĕ de Angelis, M. (2020). DLL1‐ and DLL4‐mediated Notch signaling is essential for adult pancreatic islet homeostasis. Diabetes, 69, 915–926. 10.2337/db19-0795 32029480

[phy215425-bib-0058] Sansbury, B. E. , & Hill, B. G. (2014). Regulation of obesity and insulin resistance by nitric oxide. Free Radical Biology and Medicine, 73, 383–399. 10.1016/j.freeradbiomed.2014.05.016 24878261 PMC4112002

[phy215425-bib-0059] Sarkar, S. , Korolchuk, V. I. , Renna, M. , Imarisio, S. , Fleming, A. , Williams, A. , Garcia‐Arencibia, M. , Rose, C. , Luo, S. , Underwood, B. R. , Kroemer, G. , O'Kane, C. J. , & Rubinsztein, D. C. (2011). Complex inhibitory effects of nitric oxide on autophagy. Molecular Cell, 43, 19–32. 10.1016/j.molcel.2011.04.029 21726807 PMC3149661

[phy215425-bib-0060] Spears, E. , Serafimidis, I. , Powers, A. C. , & Gavalas, A. (2021). Debates in pancreatic beta cell biology: Proliferation versus progenitor differentiation and transdifferentiation in restoring β cell mass. Front Endocrinol (Lausanne), 12, 722250. 10.3389/fendo.2021.722250 34421829 PMC8378310

[phy215425-bib-0061] Stehouwer, C. D. A. (2018). Microvascular dysfunction and hyperglycemia: A vicious cycle with widespread consequences. Diabetes, 67, 1729–1741. 10.2337/dbi17-0044 30135134

[phy215425-bib-0062] Vecoli, C. , Andreassi, M. G. , Liga, R. , Colombo, M. G. , Coceani, M. , Carpeggiani, C. , L'Abbate, A. , & Neglia, D. (2012). T(−786*)*→C polymorphism of the endothelial nitric oxide synthase gene is associated with insulin resistance in patients with ischemic or non‐ischemic cardiomyopathy. BMC Medical Genetics, 13, 92. 10.1186/1471-2350-13-92 23031426 PMC3495192

[phy215425-bib-0063] Vecoli, C. , Novelli, M. , Pippa, A. , Giacopelli, D. , Beffy, P. , Masiello, P. , L'Abbate, A. , & Neglia, D. (2014). Partial deletion of eNOS gene causes hyperinsulinemic state, unbalance of cardiac insulin signaling pathways and coronary dysfunction independently of high fat diet. PLoS One, 9, e104156–e104166. 10.1371/journal.pone.0104156 25093405 PMC4122412

[phy215425-bib-0064] Wang, G. S. , Kauri, L. M. , Patrick, C. , Bareggi, M. , Rosenberg, L. , & Scott, F. W. (2010). Enhanced islet expansion by beta‐cell proliferation in young diabetes‐prone rats fed a protective diet. Journal of Cellular Physiology, 224, 501–508. 10.1002/jcp.22151 20432450

[phy215425-bib-0065] Wang, X. L. , Zhang, L. , Youker, K. , Zhang, M. X. , Wang, J. , LeMaire, S. A. , Coselli, J. S. , & Shen, Y. H. (2006). Free fatty acids inhibit insulin signalling‐stimulated endothelial nitric oxide synthase activation through upregulating PTEN or inhibiting Akt kinase. Diabetes, 55, 2301–2310. 10.2337/db05-1574 16873694

[phy215425-bib-0066] Wang, Y. , Fang, Y. , Lu, P. , Wu, B. , & Zhou, B. (2021). NOTCH signaling in aortic valve development and calcific aortic valve disease. Frontiers in Cardiovascular Medicine, 8, 682298. 10.3389/fcvm.2021.682298 34239905 PMC8259786

[phy215425-bib-0067] Xu, X. , D'Hoker, J. , Stangé, G. , Bonné, S. , De Leu, N. , Xiao, X. , Van de Casteele, M. , Mellitzer, G. , Ling, Z. , Pipeleers, D. , Bouwens, L. , Scharfmann, R. , Gradwohl, G. , & Heimberg, H. (2008). Beta cells can be generated from endogenous progenitors in injured adult mouse pancreas. Cell, 132, 197–207. 10.1016/j.cell.2007.12.015 18243096

[phy215425-bib-0068] Zhang, T. , Guo, L. , Wang, Y. , & Yang, Y. (2018). Macroautophagy regulates nuclear NOTCH1 activity through multiple p62 binding sites. IUBMB Life, 70, 985–994. 10.1002/iub.1891 30207627 PMC6153043

[phy215425-bib-0069] Zhang, X. , Tao, J. , Yu, J. , Hu, N. , Zhang, X. , Wang, G. , Feng, J. , Xiong, X. , Li, M. , Chai, D. , Li, H. , Rong, Y. , Tang, Z. , Wang, W. , Peng, Z. , & Shi, Q. (2021). Inhibition of Notch activity promotes pancreatic cytokeratin 5‐positive cell differentiation to beta cells and improves glucose homeostasis following acute pancreatitis. Cell Death and Disease, 12, 867. 10.1038/s41419-021-04160-2 34556631 PMC8460737

[phy215425-bib-0070] Zhou, B. , Lin, W. , Long, Y. , Yang, Y. , Zhang, H. , Wu, K. , & Chu, Q. (2022). Notch signaling pathway: Architecture, disease, and therapeutics. Signal Transduction and Targeted Therapy, 7, 95. 10.1038/s41392-022-00934-y 35332121 PMC8948217

[phy215425-bib-0071] Zhou, Q. , Brown, J. , Kanarek, A. , Rajagopal, J. , & Melton, D. A. (2008). In vivo reprogramming of adult pancreatic exocrine cells to beta‐cells. Nature, 455(7213), 627–632. 10.1038/nature07314 18754011 PMC9011918

